# Human Cell Line-Derived Monoclonal IgA Antibodies for Cancer Immunotherapy

**DOI:** 10.3390/bioengineering4020042

**Published:** 2017-05-08

**Authors:** Felix Hart, Antje Danielczyk, Steffen Goletz

**Affiliations:** Glycotope GmbH, Robert-Roessle-Street 10, 13125 Berlin, Germany; antje.danielczyk@glycotope.com (A.D.); sgoletz@web.de (S.G.)

**Keywords:** IgA antibody, cancer therapy, human expression system, human glycosylation, glyco-optimization, perfusion process, TA-mucin 1, Her2, EGFR, Thomsen–Friedenreich, CD20

## Abstract

IgA antibodies have great potential to improve the functional diversity of current IgG antibody-based cancer immunotherapy options. However, IgA production and purification is not well established, which can at least in part be attributed to the more complex glycosylation as compared to IgG antibodies. IgA antibodies possess up to five *N*-glycosylation sites within their constant region of the heavy chain as compared to one site for IgG antibodies. The human GlycoExpress expression system was developed to produce biotherapeutics with optimized glycosylation and used here to generate a panel of IgA isotype antibodies directed against targets for solid (TA-mucin 1, Her2, EGFR, Thomsen–Friedenreich) and hematological (CD20) cancer indications. The feasibility of good manufacturing practice was shown by the production of 11 g IgA within 35 days in a one liter perfusion bioreactor, and IgA antibodies in high purity were obtained after purification. The monoclonal IgA antibodies possessed a high sialylation degree, and no non-human glycan structures were detected. Kinetic analysis revealed increased avidity antigen binding for IgA dimers as compared to monomeric antibodies. The IgA antibodies exhibited potent Fab- and Fc-mediated functionalities against cancer cell lines, whereby especially granulocytes are recruited. Therefore, for patients who do not sufficiently benefit from therapeutic IgG antibodies, IgA antibodies may complement current regiment options and represent a promising strategy for cancer immunotherapy. In conclusion, a panel of novel biofunctional IgA antibodies with human glycosylation was successfully generated.

## 1. Introduction

Today, all approved monoclonal antibodies for cancer therapy are immunoglobulin (Ig) G isotype antibodies [1,2]. Methods for IgG production and purification are well known, and this isotype benefits from prolonged serum half-life through neonatal Fc receptor-mediated recycling. However, several studies indicate better cytotoxic effects against cancer cells in vitro when engaging FcαRI on granulocytes. Using granulocytes as the source of effector cells, FcαRI-engagement can induce potent antibody-dependent cellular cytotoxicity (ADCC) against tumor cells [3,4,5,6,7,8,9,10].

IgA is the predominant immunoglobulin in mucosal secretions, where it serves as first line of defense and protects against pathogens that are ingested or inhaled [11,12]. In human serum, IgA is the second most prevalent antibody and serves as second line of defense. Serum IgA is mainly monomeric, while secretory IgA (SIgA) is dimeric and associated with additional polypeptides, the joining (J) chain and secretory component (SC) [12,13,14,15,16]. Per day, an adult produces about 66 mg IgA antibodies per kilogram bodyweight, which is more than all other isotypes combined [17].

**Figure 1:**
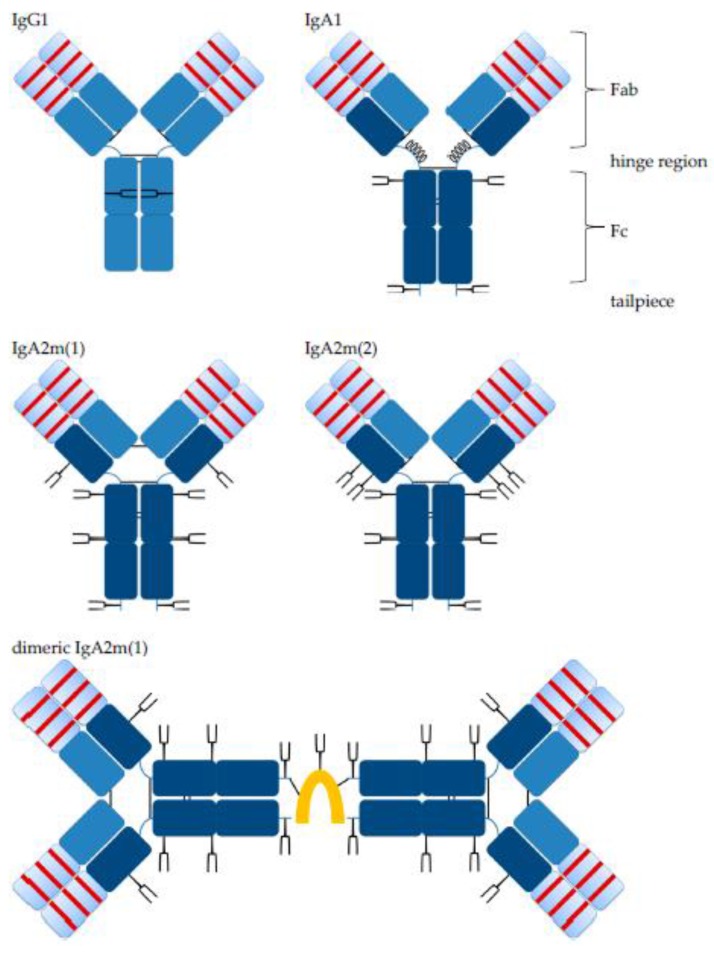
Schematic illustration of IgG1 and IgA antibodies. IgG Cγ and common Cκ light chain domains (both blue) and IgA Cα domains (dark blue) are indicated. Variable domains (light blue) including complementary-determining regions (CDRs, red bars) of heavy and light chains are indicated. *N*-glycosylation sites (*Y*) with their different orientation towards the inside or outside of the molecules are indicated for IgG or IgA, respectively. Antibodies might possess *N*-glycosylation sites within their variable domain, which is not shown here. The tailpiece for IgA antibodies is shown, which is not present for IgG antibodies. Inter-chain disulfide bonds (black lines) of heavy and light chains are indicated and differ between isotypes and allotypes. In contrast to IgA2, in the extended hinge region of IgA1, *O*-glycosylation sites (0) are present. IgA2m(1) forms disulfide bonds between the two light chains. Dimeric IgA2m(1) include inter-chain disulfide bonds between the J chain (orange) and two IgA monomer units. The J chain stabilizes IgA dimers, but other interactions between IgA monomer units can result in the formation of dimers lacking the J chain. Adopted from Woof and Burton [18].

Structurally, IgA and IgG antibody monomer units consist of two fragment antigen binding (Fab) domains, including the variable antigen binding part and the constant fragment crystallizable (Fc) domain, which binds to different receptors, including those of immune effector cells and other components of the immune system, and a connecting hinge region (Figure 1). 

Like IgG, IgA monomers consist of two light chains (LC, kappa or lambda) and two heavy chains (HC). The HC contains a variable region (VH) and three constant regions, Cα1, Cα2 and Cα3. In humans, there are two alpha HC genes, encoding IgA1 or IgA2 subclasses [12,19]. Both, IgA1 and IgA2 HC contain an 18-amino acid C-terminal tailpiece with a penultimate Cys residue, which is found on IgM antibodies, as well. There is one allotype for IgA1, whereas three allotypes for IgA2 were described: IgA2m(1), IgA2m(2) and IgA2(n) [20,21]. Of note, IgA2m(1) lacks disulfide bonds between HCs and LCs, which is found in other isotypes; instead, the two LCs are linked by disulfide bonds, and the HC-LC interactions are non-covalent [14]. IgA1 possesses a 23-amino acid hinge region, while the hinge region of IgA2 is 10 amino acids in length. The extended hinge region is rich in proline, serine and threonine and carries two to five, in some cases up to six, *O*-glycosylations [22,23,24]. Each *O*-glycosylation at a serine or threonine in the hinge region can be composed of *N*-acetylgalactosamine, galactose and sialic acid in a heterogenic mixture [19,23,25]. Apart from the *O*-glycosylation, IgA1 has two *N*-glycosylation sites per heavy chain. For IgA2m(1), there are two additional *N*-glycosylation sites, and IgA2m(2) possesses a fifth *N*-glycosylation site. Most of the *N*-glycans are biantennary complex-type structures with a minority of triantennary and tetra-antennary glycans, as well as oligomannose structures [19,26]. In contrast to IgG1 where glycans are orientated towards the inside between the two Cγ2 domains, molecular modeling revealed a IgA glycan orientation towards the outside of the molecule [24]. Compared to IgG, IgA glycosylation is characterized by more complete processing, potentially because of better accessibility of the glycans for glycosyltransferases within the Golgi. Hence, the degree of sialylation is much higher for IgA than for IgG, 90% and 10%, respectively [24]. A putative role of IgA glycosylations is protection against self-aggregation [27]. For IgG antibodies, the Fc glycosylation is important for effective Fc gamma receptor (FcγR) binding and biofunctionality. Affinity to FcγRs is decreased by deglycosylation or certain alterations like increased sialylation in the glycan structures, while reduced core-fucosylation enhances ADCC and antibody-dependent cellular phagocytosis (ADCP) killing activities mediated by natural killer (NK) cells or macrophages against tumor cells [28,29,30,31,32]. In the case of IgA, deletion of IgA1 glycosylation motifs had no effect on binding to its main receptor, Fc alpha receptor I (FcαRI; CD89) [24]. 

While IgG antibodies are only present as monomers, IgA antibodies can form dimers and higher order polymers by themselves or in complex with additional components. Mucosal plasma cells co-express the J chain, a 15 kDa polypeptide, which associates with IgA dimers. A disulfide bridge is formed between J chain and Cys residues of each IgA antibody. The J chain has one *N*-glycosylation site and contains three intra-chain disulfide bonds. Two additional disulfide bonds are formed between the J chain and two IgA monomer units within the tailpiece (Figure 1). The same polypeptide associates with pentameric IgM antibodies. A second polypeptide associates with SIgA; the SC is about 80 kDa in size and the extracellular part of the polymeric immunoglobulin receptor (pIgR). During epithelial cell transcytosis mediated by the pIgR, the SC is cleaved off at the apical site to release SIgA, which consists of two IgA monomers, one J chain and one SC [14,33]. 

The major receptor binding IgA is FcαRI. It is expressed on neutrophils, eosinophils, monocytes, macrophages, interstitial dendritic cells and Kupffer cells [17]. Differences in structural binding orientation might allow a stoichiometry for FcαRI and IgA binding of 2:1 as compared to a 1:1 stoichiometry for FcγRIII and IgG [18,34,35]. 

Depending on the effector cell, activation of the FcαRI can lead to multiple cellular functionalities including ADCP, ADCC, respiratory burst, degranulation, cytokine release and antigen-presentation [33,36]. However, upon monovalent binding (e.g., by serum IgA, which is not present as an immune complex), the FcαRI can also mediate inhibitory signals [17,37]. Other activating receptors co-locate within lipid rafts with the FcαRI inhibitory complex. Therefore, the IgA receptor can circumvent excessive immune responses by inhibiting other activating receptors [38,39]. 

Neutralizing and inflammatory functions are described for IgA antibodies, but at the same time, IgA antibodies also represent inflammatory regulators to prevent excessive immune responses [36]. Taken together, these diverse functions could be harnessed to use tumor-specific IgA antibodies for cancer immunotherapy. Additionally, IgA antibodies were also investigated to treat infection diseases. One of the best known functions of IgA antibodies is immune exclusion. Indeed, IgA antibodies showed efficacy against tuberculosis [40] and in a murine malaria model [41]. Targeting influenza virus hemagglutinin using IgG or IgA antibodies revealed higher potential to inhibit virus particle release from infected cells by IgA antibodies. This might have been caused by increased avidity since polymeric IgA was compared with monomeric IgG antibodies [42]. Together, these studies indicate that therapeutic IgA antibodies might be applied to sites where IgG antibodies cannot be administered or when bivalent binding of IgG antibodies is not sufficient for successful immune exclusion of pathogens.

However, although structural and functional characteristics of IgA antibodies and their potential for clinical use are widely explored, IgA antibodies are not yet established as therapeutics for human use. One drawback is the challenging in vivo efficacy evaluation of therapeutic IgA antibodies due to the lack of an IgA receptor homologue in mice and the relatively short serum half-life [13,43]. Moreover, there is a lack of efficient production systems with high productivity and reproducibility. The production and purification of monoclonal IgA antibodies is not well established [9,44]. IgA production rates and yields using conventional expression systems are often low, and assembly is sometimes incomplete [8,45,46,47]. The high number of glycosylation sites potentially results in more heterogeneous recombinant products as compared to IgG antibodies. IgA antibody glycans are surface exposed and involved in pathological conditions, which highlights the importance of human-like IgA production [19,23,24,25,48,49,50]. Today, different expression systems are used for the production of therapeutic antibodies, which in most cases are rodent cells. For example, Mabthera, Gazyvaro, Vectibix and Herceptin are produced in Chinese hamster ovary (CHO) cells, and Erbitux is produced in murine Sp2/0 cells [51]. While rodent and human expression systems share common characteristics, some distinct features can affect safety and efficacy of therapeutic proteins. Structures that are absent in human glycans like α(1-3)-linked galactose or α(2-3)-linked *N*-glycolylneuraminic acid are potentially immunogenic for patients [29]. Moreover, CHO cells are not capable of linking *N*-acetylneuraminic acid in α(2-6) configuration, which is present in human glycans [29]. These limitations of commonly used non-human expression systems can be overcome by fully-human systems like the myelogenous leukemia-derived GlycoExpress (GEX) cell lines. In addition to the fully-human glycans, glycoengineering was used to generate cell lines that produce biologics with specific glycosylation patterns. For example, GEX cell lines are used for the production of proteins with high sialylated glycans or high sialylated glycans with low fucose levels. The reduction in core fucose of *N*-glycans within the Cγ2 domain of IgG antibodies results in increased FcγRIIIa affinity and Fc-mediated effector functionality [31,52]. 

In order to elucidate the potential of IgA isotype antibodies for cancer immunotherapy, antibodies directed against five heterogeneous targets were generated. The antibodies are specific for epidermal growth factor receptors, glyco-peptide and glycan epitopes, as well as a surface marker for B cells. This broad spectrum of IgA antibodies was biofunctional against cancer cell lines. IgA1 and IgA2 antibodies, as well as IgA antibodies in association with the J chain to increase binding avidity were expressed. In order to account for the importance of optimal human-like glycosylation, the fully-human GEX expression system was used for antibody production. 

## 2. Materials and Methods 

### 2.1. Chemicals and Consumables

Unless otherwise noted, chemicals were purchased from Carl Roth (Karlsruhe, Germany), Merck KGaA (Darmstadt, Germany) or Sigma-Aldrich Chemie GmbH (Steinheim, Germany). Additional chemicals and consumables are specified in Supplementary B. 

### 2.2. Recombinant Antibody Production

DNA sequences encoding variable light or heavy chains were purchased from Geneart and supplied in a plasmid with ampicillin resistance. DNA cloning was conducted according to standard protocols [53]. All IgA2 antibodies were of the IgA2m(1) allotype. Sequences are listed in Supplementary B. If not otherwise noted, all eukaryotic cells were grown at 37 °C, 5% CO_2_ and 90% relative humidity. The mAbExpress GEX cell line was developed by Glycotope (Berlin, Germany) and used for antibody production. 

Nucleofector kit V (Lonza, Basel, Switzerland) was used for stable transfections. For electroporation, 2 × 10^6^ cells with >90% vitality were resuspended in 100 μL Nucleofector solution containing expression plasmids and transferred into a cuvette. A Nucleofector II instrument (Amaxa, Cologne, Germany) was used for electroporation, and after two days, selection pressure media was used for cultivation. 

At a selection pressure of 100 nM methotrexate and 0.4 μg/mL puromycin, clones with high antibody production were selected using CloneMatrix with anti-IgG (H+L) CloneDetect (both Molecular Devices, Sunnyvale, CA, USA) for cultivation. High producing clones were picked based on total exterior fluorescence using a ClonePix instrument (Molecular Devices). Subsequent selections of clones were based on specific production rate or maximal titers within 3 or 4 days. Usually, 3–5 clones per antibody were kept for antibody production and preserved by cryogenic conservation.

For J chain co-expression, high producing clones were used for subsequent transfection with a J chain-encoding plasmid. After transfection, clones were screened for J chain expression by anti-J chain Western blots. 

Antibodies were produced in 250 mL to 1 L spinner culture flasks over 3–5 days. Prior to storage at −20 °C, supernatants were filtered using a 1 μm glass fiber filter membrane (Pall). 

### 2.3. Eukaryotic Cell Lines

The following target cells were used: A-431, BT-474, Panc-1, Raji, SK-BR-3 and ZR-75-1. Media compositions, sources and media supplements are listed in Supplementary B. Unless otherwise noted, cells were centrifuged at ~300× *g* for 5 min. The Quantum Simply Cellular anti-human IgG kit (Bangs Laboratories, Fishers, IN, USA) was used according to the manufacturer’s protocol. IgG isotype antibodies were used to determine the number of binding sites and to classify target cells according to the number of antigen binding sites: + (1 × 10^4^ to 1 × 10^5^), ++ (1 × 10^5^ to 5 × 10^5^) or +++ (>5 × 10^5^).

### 2.4. Perfusion Process in the 2-L Stirred Tank Bioreactor

For inoculation to a final cell concentration of 2 × 10^5^ viable cells/mL, cells were grown in spinner culture. The working volume was 1 L using a 2 L stirred tank bioreactor (BBI Biotech Quad System, Berlin, Germany) and the following process parameters: 37 °C, 40% dissolved oxygen, pH 7.0, 300–400 rpm stirrer speed depending on dissolved oxygen. During the process, medium was continuously fed while harvesting supernatant discontinuously using a Centritech Lab II (Barry-Wehmiller, PneumaticScaleAngelus, Stow, OH, USA) in intermittent mode. The wait time between cycles was adjusted to control the perfusion rate and thereby keeping a constant working volume of 1 L. 

When glucose dropped below 2.5 g/L (usually by Day 4 or 5), continuous operation was started by feeding Glycotope medium at a perfusion rate of 0.25 V/d. Perfusion was stepwise increased up to 2 V/d when glucose concentration dropped below 2.5 g/L or every other day. When the maximum perfusion rate was reached, feed media was gradually replaced by enriched two-times concentrated Glycotope medium. 

### 2.5. Antibody Purification by Affinity Chromatography

Culture supernatants or bioreactor harvests were filtered using 0.22 μm bottle top filters (TPP, Trasadingen, Switzerland). For purification, 1 mL KappaSelect columns (GE Healthcare, Little Chalfont, United Kingdom) were used with an ÄKTAPrime (GE Healthcare) or fast protein liquid chromatography (FPLC) system. Phosphate-buffered saline (PBS) served as running and washing buffer, and the flow rate was set to 1 mL/min. Chromatography was carried out at ambient temperature while supernatants were kept on ice during sample loading. Antibodies were eluted with 10 CV 0.1 M glycine pH 2.5, and 1 mL fractions were collected in 1.5 mL centrifuge tubes containing 200 μL 2.45 M potassium phosphate buffer pH 7.2 for neutralization. Fractions containing protein according to UV absorbance at 280 nm were pooled. Buffer exchange of the solution into PBS was carried out by concentrating and diluting using 50,000 molecular weight cut off Amicon tubes (Merck Millipore, Billerica, MA, USA). For adequate buffer exchange, this step was repeated at least 7 times, followed by 0.22 μm filtration using a syringe filter. Protein concentration was determined using a NanoDrop 2000c (Thermo Fisher Scientific, Waltham, MA, USA) spectrophotometer. Antibodies were stored at 4 °C. 

### 2.6. Sodium Dodecyl Sulfate Polyacrylamide Gel Electrophoresis and Western Blots

SDS-PAGE and Western blots were performed according to standard protocols [53], loading 3 μg and 0.5 μg for each sample per lane, respectively. For reducing conditions, 2-mercaptoethanol was added to a final concentration of 2.5% in the samples. A Tris glycine buffer system was used with Mini-Protean TGX gels (Bio-Rad, Hercules, CA, USA) and a Mini-Protean Tetra system (Bio-Rad). Proteins were visualized with colloidal Coomassie. Proteins were transferred on nitrocellulose membranes (GE Healthcare) using a Tras-Blot SD semi-dry system (Bio-Rad). 

### 2.7. Size Exclusion Chromatography

Size exclusion chromatography (SEC) was carried out on an ÄKTAPrime system (GE Healthcare) using a Superdex200 10/300 GL column (GE Healthcare) with PBS as the running buffer. As for washing and equilibration, the flow rate for analytical SEC was set to 0.5 mL/min. For preparative SEC, the flow rate was set to 0.3 mL/min, and 0.5 mL fractions were collected. Fractions corresponding to peaks in the UV absorbance signal at 280 nm were pooled. SECs were carried out at room temperature. An IgG antibody served as positive control for monomers. 

### 2.8. Surface Plasmon Resonance 

A Biacore 2000 (GE Healthcare) instrument was used for surface plasmon resonance experiments. EGFR antigen (R&D Systems) was immobilized on CM5 chips (GE Healthcare), and antibody was passed through the flow cell. The amine coupling kit (GE Healthcare) was used to immobilize EGFR (270 RU) according to the manufacture’s protocols. For the reference flow cell, ligand injection was omitted. If not otherwise noted, HBS-EP (GE Healthcare) buffer was used as the running buffer, and buffer exchange into running buffer was conducted for antibody samples. Starting concentrations were between 1500 nM and 2400 nM for IgA2 monomers and IgG antibodies. The starting concentration for IgA2 dimers was 760 nM. Seven four-fold serial dilutions and a running buffer blank were injected for each run. In kinetic experiments, a flow rate of 50 μL/min was used, and samples were injected for a 5 min association time, followed by a 17 min dissociation time. After dissociation, the chip was regenerated by a 24 s injection of 10 mM NaOH. Equilibrium dissociation constants were determined using BIAevaluation software Version 4.1 (GE Healthcare). Maximal responses were fitted locally, and curves were fitted using bivalent and tetravalent binding models (Supplementary B) for monomeric and dimeric antibodies, respectively. Equilibrium dissociation constants were calculated based on first association and dissociation rates. 

### 2.9. N-Glycan Profiling

*N*-glycans were enzymatically liberated from antibodies and labeled with 2-aminobenzamide (2-AB) for fluorescence detection and quantification employing hydrophilic interaction liquid chromatography (HILIC). Structure identification was performed based on mass spectrometry (MS). Antibodies were denatured and reduced prior to enzymatic release of *N*-glycans using PNGase F within polyacrylamide gel blocks. Liberated glycans were fluorescently labeled using the LudgerTag 2-AB (2-aminobenzamide) Glycan Labeling Kit (Ludger, Oxford, UK). Labeled glycans were separated with an ACQUITY UPLC Glycan BEH Amide Column (Waters, Milford, MA, USA). Glycan structures of each peak were identified by MS. Therefore, either MALDI-TOF MS/post source dissociation MS (Microflex, Bruker; Billerica, MA, USA) of fractionated *N*-glycans co-crystallized with dihydroxybenzoic acid or online LC-ESI-qTOF CID-MS/MS (Impact HD, Bruker) was used. Glycan structures of each peak were then analyzed using a combination of MS and MS/MS fragmentation data with the Compass DataAnalysis software 4.3 (Bruker). Quantification was based on fluorescence signals using the Empower 3 software (Waters). 

### 2.10. Flow Cytometry

If not otherwise noted, 1 × 10^5^ to 2 × 10^5^ cells were used for flow cytometry analyses using a BD FACSCanto II (BD Biosciences, San Jose, CA, USA) in 96-well plate format. Staining with primary labeled antibodies, non-labeled antibodies or labeled secondary antibodies (Supplementary B) was conducted in 50 μL PBS containing 5% heat-inactivated fetal bovine serum (Biochrom) for 20–30 min at 4 °C. Subsequently, cells were washed twice with PBS and resuspended in 100 μL for flow cytometry analyses. For data evaluation, BD FACSDiva software Version 8.0 (BD Biosciences) was used.

### 2.11. Proliferation Inhibition

For proliferation inhibition of target cells, 5000 cells in 75 μL medium were transferred in 96-well flat bottom plates. Subsequently, 75 μL two-fold concentrated antibody diluted in medium were added to the cells. After 4–5 days incubation at 37 °C, 5% CO_2_ in a humidified incubator, metabolic active cells were determined using the CellTiter-Glo Luminescent Cell Viability Assay (Promega) kit according to the manufacturer’s protocol. Proliferation was expressed relative to a control grown in the absence of antibody. Ten micromoles of aphidicolin served as a positive control for proliferation inhibition. 

### 2.12. Antibody-Dependent Cellular Cytotoxicity Assay

ADCC assays were based on antibody concentration-dependent europium release of target cells in the presence of granulocytes as effector cells. Peripheral blood was drawn from healthy donors using a heparin-coated Vacutainer (BD Biosciences). Nine milliliters of blood were layered on top of 20 mL 1.079 g/mL Easycoll solution (Biochrom) diluted in PBS using 50 mL conical centrifuge tubes. After a centrifugation at 400× *g* for 30 min with minimal acceleration and break settings, the upper phase, the interphase and the upper Easycoll phase were discarded. The tubes were filled up to about 47 mL with Pharm Lyse (BD Biosciences), mixed thoroughly and incubated for 15 min at room temperature. After centrifugation at 600× *g* for 5 min, the supernatant was discarded, and the granulocyte pellets were pooled in one 50 mL conical centrifugal tube. Subsequently, two wash steps with PBS were conducted, and granulocytes were seeded in Roswell Park Memorial Institute (RPMI) 1640 medium (Biochrom) containing 2 mM l-glutamine (Biochrom), 10% heat-inactivated fetal bovine serum (Biochrom) and 100 U/mL Interferon γ (Miltenyi Biotec, Bergisch Gladbach, Germany) at 1 × 10^6^ cells/mL. Granulocytes were used in ADCC assays after overnight incubation at 37 °C, 5% CO_2_ in a humidified incubator.

Prior to europium loading, target cells were washed with PBS, and 3 × 10^6^ target cells were resuspended in 100 μL cold europium buffer containing 50 mM N-2-hydroxyethylpiperazine-N'-2-ethane sulfonic acid (HEPES, Carl Roth), 93 mM sodium chloride (Carl Roth), 5 mM potassium chloride (Carl Roth), 2 mM magnesium chloride (Merck Millipore), 10 mM diethylenetriaminepentaacetic acid (Sigma-Aldrich), 2 mM europium-III acetate (Sigma-Aldrich) pH 7.4. After 10 min incubation on ice, target cells were electroporated using a Nucleofector II (Amaxa), followed by another 10 min incubation on ice. Then, target cells were gently pipetted into 13 mL RPMI 1640 medium containing 5% (*v*/*v*) heat-inactivated fetal bovine serum (Biochrom, Berlin, Germany) and centrifuged. This wash step was repeated six times before pipetting target cells in the assay plate. 

ADCC assays were conducted in round bottom 96-well plates. Twenty microliters of ten-fold concentrated antibody dilutions in RPMI 1640 medium containing 5% (*v*/*v*) heat-inactivated fetal bovine serum (Biochrom) or controls were transferred into plates. Next, 100 μL of 5 × 10^4^ europium-loaded washed target cells per milliliter were added. Then, 80 μL of 5 × 10^6^ effector cells per milliliter were added, and plates were incubated for 3–5 h at 37 °C, 5% CO_2_ in a humidified incubator. After incubation, plates were centrifuged, and 20 μL supernatant were transferred into white 96-well plates containing 200 μL enhancement solution (PerkinElmer, Waltham, MA, USA). After a 10 min incubation in the dark, fluorescence was measured using a microplate reader (Tecan, Männedorf, Switzerland). Samples and controls were analyzed in triplicates and sextuplicates, respectively. Target cells in the absence of effector cells and antibody served as spontaneous release controls. Specific lysis was calculated relative to the maximal europium release using 1% (*v*/*v*) Triton X-100 (Sigma-Aldrich) in the absence of effector cells and antibody as specified in Equation (1):

(1) $$specific\;lysis\;\left(\%\right)=\;\frac{signal_{sample}-mean\;signal_{spontaneous\;release}\;}{mean\;signal_{maximal\;release}-mean\;signal_{spontaneous\;release}}\times 100\%$$

### 2.13. Antibody-Dependent Cellular Phagocytosis Assay

Buffy coats served as source for monocytes, which were subsequently differentiated into macrophages. Monocytes were isolated by magnetic sorting using the Pan Monocyte Isolation Kit and LS columns (both Miltenyi Biotec) according to the manufacturer’s protocol. For macrophage differentiation, 6 × 10^5^ monocytes per mL were cultured over six to seven days in AIM-V medium (Thermo Fisher Scientific) containing 2 mM l-glutamine (Biochrom), 25 mM HEPES (Carl Roth), 50 μM 2-mercaptoethanol (Sigma-Aldrich), 2% (*v*/*v*) heat-inactivated human AB serum (Sigma-Aldrich), 10 mM 1,25-dihydroxyvitamin D3 (Sigma-Aldrich) at 37 °C, 5% CO_2_ in a humidified incubator. After the first day, cultivation was continued in the presence of 500 U/mL GM-CSF (Peprotech, Rocky Hill, NJ, USA). During the cultivation, fresh medium was supplied every two to three days. The day before the phagocytosis assays, target cells were labeled with the fluorescent dye PKH26 (Sigma-Aldrich) according to the manufacturer’s instructions. 

The day of phagocytosis assays, monocyte-derived macrophages (MDM) were harvested with trypsin and adjusted to 1 × 10^6^ cells/mL in medium. Using low binding 96-well round bottom plates (Nunc), 100 μL MDM were used, and 50 μL of harvested target cells at a concentration of 4 × 10^5^ cells/mL were added. Subsequently, 40 μL of samples or controls were added. For inhibition of phagocytosis, 10 μL cytochalasin D (Enzo) to yield a final concentration of 2.5 μg/mL was added. Otherwise, 10 μL PBS were used in samples for phagocytosis. After incubation for 3 h to 4 h at 37 °C, 5% CO_2_ in a humidified incubator, cells were centrifuged at ~490× *g* for 5 min and washed with PBS. Next, cells were stained for 15 min at 4 °C with a mixture of APC-labeled anti-CD45 antibody and 7-aminoactinomycin D (7-AAD) to exclude dead cells using 3 μL per well and a final concentration of 20 μg/mL, respectively. After a wash step with PBS, cells were resuspended in 100 μL, and 60 μL were analyzed by flow cytometry. The following compensations were used to account for overlapping fluorescence signals: 7-AAD to PE 18.78, PE to 7-AAD 9.57, APC to 7-AAD 20.93 and 7-AAD to APC 0.28. Percent phagocytosis was calculated based on CD45/PKH26 double-positive cells relative to CD45-positive cells. 

### 2.14. B Cell Depletion in Whole Blood

B cell depletion in whole blood was adapted from Moessner et al. [54]. Whole blood from healthy donors withdrawn in a heparin-coated Vacutainer (BD Biosciences) was used in B cell depletion assays. Twenty microliters of ten-fold concentrated antibody were added to 180 μL whole blood in 96-well round bottom plates. After incubation, 40 μL whole blood were added to a mixture of 5 μL FITC-labeled anti-CD3 and 5 μL APC-labeled anti-CD19 antibodies (both BD Biosciences) in 96-well round bottom plates. After incubation for 20 min at 4 °C, 200 μL Pharm Lyse buffer (BD Biosciences) were added for erythrocyte lysis. After a 20-min incubation at room temperature, the plates were centrifuged, and the supernatant was discarded. The lysis was repeated, and after centrifugation, the cell pellet was resuspended in 100 μL PBS for flow cytometry analysis using a BD FACSCanto II (BD Biosciences) and BD FACSDiva software Version 8.0 (BD Biosciences) for data evaluation. CD3 and CD19 served as marker for T and B cells, respectively. B cell counts relative to control T cell counts were evaluated, and B cell depletion was calculated according to Equation (2).

(2) $$B\;cell\;depletion\;\left(\%\right)=100\%-\;\frac{{\left(B\;cell\;count/T\;cell\;count\right)}_{sample}}{mean{\left(B\;cell\;count/T\;cell\;count\right)}_{medium\;controls}}\times 100\%.$$

### 2.15. Software and Statistical Analysis

Unless otherwise noted, GraphPad Prism 5 (GraphPad Software, La Jolla, CA, USA) was used to prepare graphs and for statistical analysis. 

## 3. Results

### 3.1. Generation of Human Cell Line-Derived Monoclonal IgA Antibodies Directed against Five Targets for Cancer Therapy 

The human GlycoExpress (GEX) expression system was used to generate fully-human recombinant IgA antibodies. After single cell cloning, specific production rate screenings served as the selection criterion between different clones. Differences in antibody production were observed between antibodies targeting different antigens. For example, expression levels in standard spinner culture of hTM IgA2 were noticeably higher than for CM IgA2, 49.77 mg/L–59.7 mg/L and 5.87 mg/L–7.2 mg/L, respectively. In the case of hPM, IgA1 expression levels were higher than for IgA2, 42 mg/L and 4 mg/L, respectively (Table 1). 

A panel of IgA antibodies against five different tumor antigens was successfully expressed. In all cases, production yields using serum-free media were sufficient for subsequent purification, biochemical assessment and confirming biofunctionality against cancer cell lines. Nevertheless, to evaluate the feasibility to produce large amounts of monoclonal IgA antibodies, cells secreting hTM IgA2 were cultivated in a bioreactor under serum-free conditions. 

#### High-Yield Production of hTM IgA2 by Cultivation in a Bioreactor

**Figure 2:**
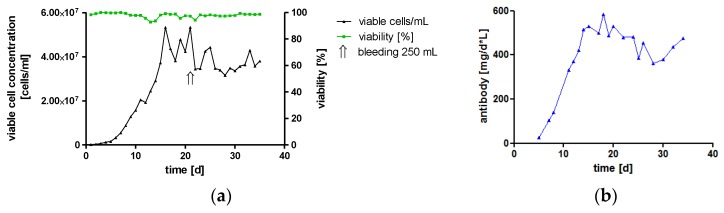
hTM IgA2 was produced in high yield by a perfusion process in a bioreactor. The working volume was 1 L using a 2 L stirred tank bioreactor. A Centritech was used for supernatant harvest and cell retention. (**a**) Viable cell concentration (black) and viability (green) during the 35-day process is shown. To maintain cell concentrations blow 5 × 10^7^ viable cells/mL, 250 mL were removed once from the bioreactor (indicated by arrow). (**b**) Productivity (blue) was calculated based on titer enzyme-linked immunosorbent assays (ELISA) values multiplied by the perfusion rate and the working volume.

One major challenge of therapeutic IgA antibodies is the production of sufficient amounts of recombinant protein [9]. To investigate the feasibility of high-yield production under conditions compatible with large-scale good manufacturing practice (GMP) production, hTM IgA2-producing cells were cultivated in a 2 L bioreactor. In the controlled environment using a continuous perfusion process with serum-free medium, high cell densities were reached. Maximal cell concentration was 5 × 10^7^ viable cells/mL, which is about ten-times higher than maximal cell density in spinner cultures. More importantly, once the culture had reached the maximal perfusion rate, IgA titers reached up to 282 μg/mL, and the mean titer for the duration of the perfusion process was 200 μg/mL. Therefore, at a perfusion rate of 2 L/d, the process yielded on average 400 mg IgA per day. The viability of cells was stable above 90% for the duration of the cultivation. Once, cells were removed from the bioreactor by bleeding about 25% of the reactor volume to avoid cell concentrations exceeding 5 × 10^7^ viable cells/mL and to ensure sufficient nutrient supply for the culture (Figure 2). 

This represents a continuous cultivation of an IgA-producing culture in a bioreactor. The feasibility of high yield production of recombinant monoclonal IgA antibodies was shown. The total IgA antibody yield was 11 g within 35 days of cultivation, which resembles the standard GMP manufacturing time of an IgG perfusion process. 

### 3.2. Biochemical Integrity of Novel Recombinant Monoclonal IgA Antibodies

Single-step affinity chromatography specific for kappa LC allowed for purification of all IgA antibodies from culture supernatants. Purity and identity were confirmed by SDS-PAGE and Western blots, respectively. Size exclusion chromatography (SEC) served as the analytical and preparative method under native conditions. SEC was used to analyze the contents of and to prepare IgA monomers, dimers or higher order multimers. 

**Figure 3:**
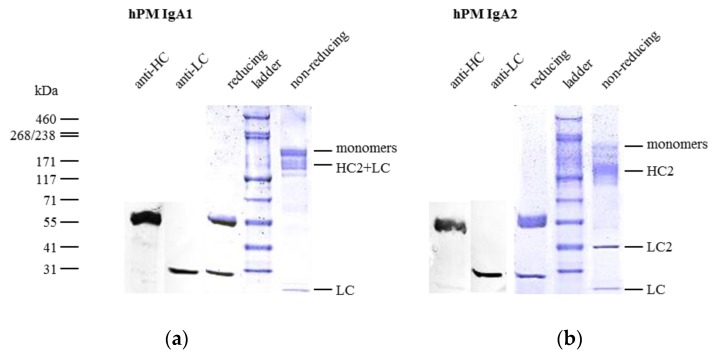
Purity assessment and comparison of affinity purified IgA1 (**a**) and IgA2 (**b**) by sodium dodecyl sulfate polyacrylamide gel electrophoresis (SDS-PAGE) and Western blots. Antibodies were analyzed using 4–15% gradient gels; in reducing conditions, only bands corresponding to the size of heavy chain (HC) and light chain (LC) were detected. Identities of bands in reducing conditions were confirmed by anti-HC and anti-LC Western blots (two left lanes). In non-reducing conditions, IgA1 antibodies showed a major band corresponding to monomers (HC2 + LC2) and minor bands for free LC and monomers lacking one LC (HC2 + LC). In contrast, IgA2 antibodies dissociated mainly in HC and LC dimers (HC2 and LC2, respectively) and LC. Bands with less intensity were detected, which corresponded to the size of IgA2 monomers or IgA2 monomers lacking one LC.

IgA1 and IgA2 antibodies differ in inter-chain disulfide bonds (Figure 1). hPM IgA1 and IgA2 were analyzed in reducing and non-reducing conditions by SDS-PAGE (Figure 3). In reducing SDS-PAGE, IgA1 (Figure 3a) and IgA2 (Figure 3b) antibodies showed comparable band patterns with two bands corresponding to the size of HC and LC. The identity of bands was confirmed by anti-human kappa LC or anti-human alpha HC Western blots (the two left bands of Figure 3a,b). No additional bands were present in reducing conditions. As estimated visually in colloidal Coomassie-stained gels, purity exceeded 95% for both IgA1 and IgA2 after single-step affinity chromatography.

In non-reducing conditions, IgA1 antibodies showed a major band corresponding to monomers (HC2 + LC2) and minor bands for free LC and monomers lacking one LC (HC2+LC) (Figure 3a). Free LC monomers could not be detected by size exclusion chromatography (SEC) where no fragments were observed (Supplementary A, Figure S1a). This indicates that some LCs were not covalently linked to IgA1 HCs.

**Figure 4:**
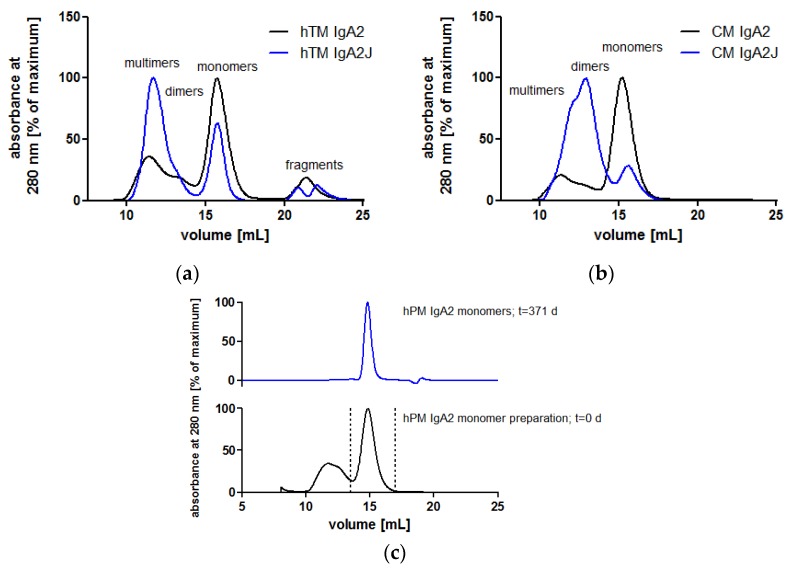
J chain co-expression promotes dimer and multimer formation of IgA antibodies, and monomers are stable during one year of storage. Comparison of hTM (**a**) and CM IgA2 (**b**) with and without J chain co-expression by size exclusion chromatography (SEC). Co-expression of the J chain resulted in a decrease of monomers and an increase in dimers and multimers. Monomer contents were 56% and 75% for hTM and CM IgA2 antibodies, respectively. Monomer contents were 27% and 16% for hTM and CM IgA2J, respectively. (**c**) hPM IgA2 monomers were generated, and after one year of storage (top, blue), no aggregation or fragmentation was observed. Fractions pooled for monomer generation by preparative SEC are indicated (vertical dotted line).

IgA2 antibodies generated for this study were of the IgA2m(1) allotype, which does not possess disulfide bonds between LC and HC, but instead between the two LCs of an IgA2 monomer unit (Figure 1). In non-reducing, but denaturing conditions of SDS-PAGE, this resulted in the formation of characteristic HC homodimers (HC2) and LC homodimers (LC2), as well as in molecules containing two HC and one LC (Figure 3b). In a small amount, LC monomers could be detected, indicating that some IgA2 LC did not form LC homodimers. The dissociation of IgA2 antibody chains was a result of SDS-PAGE conditions and not present under native conditions as used for SEC. Here, no fragments were observed (see Figure 4c). 

Both isotypes showed weak bands corresponding to the size of multimers, which were detected in SEC, as well, and were formed to some degree in the absence of the J chain (Figure 3 and Figure 4). The more diffuse bands of HCs for IgA2 antibodies compared to IgA1 might be caused by the presence of multiple *N*-glycosylation sites. IgA1 and IgA2 possess two and four *N*-glycosylation sites per HC, respectively (Figure 1). Due to a glycosylation site within the variable domain of hPM, hPM IgA1 and IgA2 possess one additional *N*-glycosylation site. These *N*-glycans can differ in both size and charge, which affects the migration behavior in SDS-PAGE.

In the absence of J chain co-expression, the produced IgA antibodies formed mainly monomers consisting of two heavy chains (HCs) and two light chains (LC). Nevertheless, IgA antibodies also associated to dimers and higher order structures as shown by SEC under native conditions (Figure 4a,b). Among all IgA antibodies without the J chain, similar SEC profiles were obtained with a major peak representing IgA monomers (Supplementary A, Figure S1). At lower elution volumes, dimers and higher order multimers were eluted. While dimers were separated from monomers, dimers and higher order structures were not well separated (Figure 4). Hence, SEC allowed for the preparation of homogeneous IgA monomers in high purity. SEC-separated hPM IgA2 monomers did not aggregate or fragment during storage at 4 °C for one year (Figure 4c). For hTM and hKM IgA2, fragments at higher elution volumes were present in SEC profiles (Figure 4a, Supplementary A, Figure S1b). The area of the IgA monomer peaks at 280 nm was 56–85% of the total area of the UV signal. The J chain polypeptide associates with IgA dimers or multimers (Figure 1). As shown for hTM and CM IgA2 antibodies by SEC, co-expression with the J chain resulted in a reduction of monomers and an increase of dimers and higher order multimers. Monomer contents of IgA antibodies without J chain were 56% and 75% for hTM and CM IgA2, respectively. By J chain co-expression, the amount of monomers decreased to 27% and 16% for hTM and CM IgA2J, respectively (Figure 4a,b). 

#### 3.2.1. Human *N*-Glycosylation Profiles of Recombinant IgA Antibodies 

Glycosylation impacts the stability, biological function and clearance of antibodies. IgA antibodies possess a more complex *N*-glycosylation than IgG antibodies and IgA *N*-glycans point towards the outside of the molecule [24,26]. IgA1 antibodies have two *N*-glycosylation sites, and IgA2m(1) antibodies have four *N*-glycosylation sites per constant region of the heavy chain as compared to one *N*-glycosylation site for IgG antibodies (Figure 1). Depending on the amino acid sequence, antibodies might have additional *N*-glycosylation sites within the variable domains as in the case of hPM and CM antibodies. 

Here, the *N*-glycan profiles for hTM, hOM, hPM, CM and hKM IgA2 antibodies were analyzed to evaluate predominant glycan structures and the degree of sialylation for recombinant IgA antibodies produced by the human GEX expression system. For the IgA antibodies analyzed, complex *N*-glycosylation profiles were obtained. All three *N*-glycan types that can be attached to proteins were identified: complex, oligomannose and hybrid type [55]. On average, 71% complex-type, 16% oligomannose and 7% hybrid-type *N*-glycans were attached to the generated IgA2 antibodies, while less than 5% could not be annotated. Among all analyzed IgA2 antibodies, the most prominent glycan structure was biantennary, core-fucosylated, digalactosylated and sialylated. Different glycan parameters were evaluated; more than 60% of complex type *N*-glycans of all antibodies were sialylated (Table 2). 

Human *N*-glycosylation was confirmed for hTM, hOM, hPM, CM and hKM IgA2 antibodies. As expected for a human expression system, non-human glycan structures like α(1-3)-linked galactose or *N*-glycolylneuraminic acid were not detected. This is in contrast to commercially available IgG antibody Erbitux, which is derived from murine Sp2/0 cells (Table 2) or antibodies derived from other non-human expression systems commonly used for the production of therapeutic proteins [29]. 

#### 3.2.2. IgA Dimers Show Increased Antigen Binding Avidity Compared to IgG Antibodies

CM IgA2 monomers and dimers were separated via SEC, and the binding kinetics of CM IgA2 monomers, dimers and CM IgG antibodies to EGFR immobilized on a CM5 chip were investigated. 

**Figure 5:**
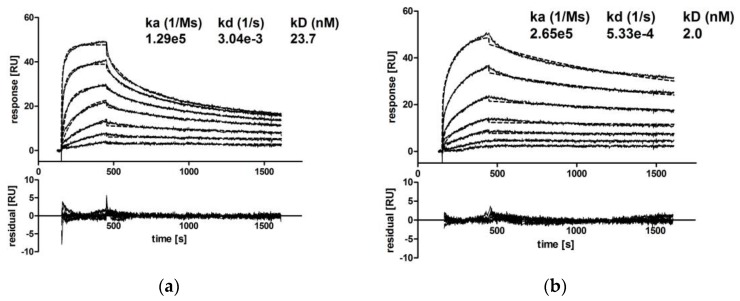

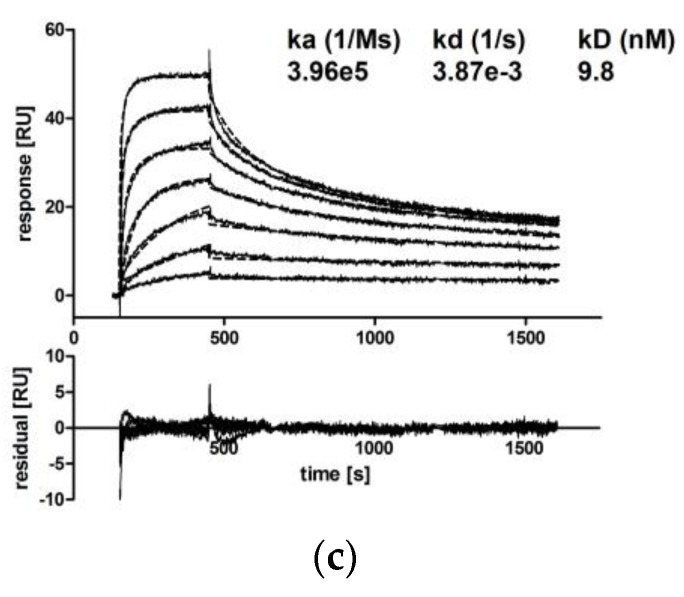
Kinetic analysis of CM IgA2 monomers, CM IgA2J dimers and CM IgG binding to recombinant EGFR. EGFR was immobilized on one flow cell of a CM5 chip, and a second flow cell served as a reference. Varying concentrations of CM IgA2 monomers (**a**), CM IgA2J dimers (**b**) or CM IgG (**c**) were injected at a flow rate of 50 μL/min for a 5 min association time and followed by a 17 min dissociation time. The difference between the flow cell with immobilized EGFR and the reference flow cell (solid lines) and fits (dotted lines) were plotted. Absolute residuals between data and fits are shown below. One out of two (IgA2 monomers and IgG) independent experiments is shown.

Due to the high dimer content, CM IgA2J was used for the preparation of dimers by repetitive preparative SECs (Supplementary A, Figure S2). Injections of multiple antibody concentrations revealed comparable binding kinetics for CM IgA2 and IgG monomers. Equilibrium dissociation constants for IgA2 and IgG were 23.7 nM and 9.8 nM, respectively, indicating high avidity binding in low nanomolar ranges (Figure 5a,c). For both isotypes, sensorgrams had similar shapes, rapid association within 5 min, followed by faster dissociation rates within 15 min as compared to CM IgA2J dimers. CM IgA2J dimers had an about 12-fold lower equilibrium dissociation constant compared to CM IgA2 monomers, 2.0 nM compared to 23.7 nM, respectively. Association of CM IgA2J dimers was slower compared to CM IgA2 monomers and IgG, while CM IgA2J dimers showed decreased dissociation rates compared to CM IgA2 monomers and IgG antibodies (Figure 5). Binding of monomeric antibodies, either IgA2 or IgG, was characterized by higher dissociation constants compared to dimeric IgA2J antibodies. This indicates an increase in avidity, which could be attributed to the higher valence of dimeric antibodies.

### 3.3. Recombinant IgA Antibodies Are Biofunctional against Cancer Target Cell Lines 

Target binding of the IgA antibodies directed against five different targets was investigated by ELISA, immunofluorescence, flow cytometry and surface plasmon resonance (Supplementary A, Figures S3–S5). Antigen and target cell binding was a prerequisite for subsequent evaluation of IgA antibodies in biofunctional assays. Binding to FcαRI confirmed the potential to recruit immune effector cells to elicit cytotoxic effects on cancer cells (Supplementary A, Figure S6). 

#### 3.3.1. Proliferation Inhibition 

**Figure 6:**
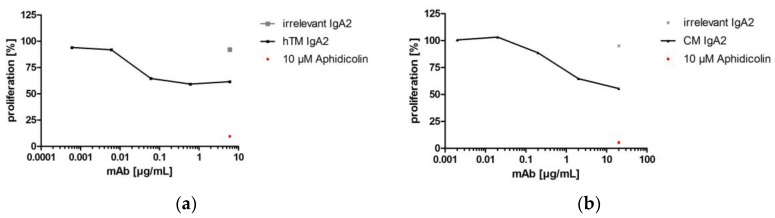
Inhibition of cancer cell line proliferation by hTM and CM IgA2 antibodies. Proliferation within 4–5 days relative to a control grown in medium without antibody was calculated. hTM IgA2 (**a**) and CM IgA2 (**b**) antibodies inhibited the proliferation of SK-BR-3 (Her2 +++) and A-431 (EGFR ++) target cells, respectively. For SK-BR-3 and A-431, hOM IgA2 antibody served as the irrelevant matched isotype negative control. One representative out of three independent experiments is shown. Mean values of duplicates are shown. Antigen binding sites were determined using IgG antibodies; antigen binding sites: +, 1 × 10^4^ to 1 × 10^5^; ++, 1 × 10^5^ to 5 × 10^5^; +++, >5 × 10^5^.

One important Fab-mediated effector function of antibodies targeting epidermal growth factor receptors like EGFR or Her2 is the prevention of uncontrolled cancer cell proliferation [56,57]. By EGFR binding, antibodies like cetuximab prevent binding of activating ligands and thereby inhibit proliferation. Trastuzumab prevents proliferative signaling, which is initiated by homo- and hetero-dimerization of its target Her2. Upon binding to Her2, trastuzumab inhibits phosphorylation of cytoplasmic kinases and thereby proliferative signaling of this receptor [58]. Inhibition of SK-BR-3 or A-431 cancer target cell line proliferation in the presence of hTM and CM IgA antibodies targeting Her2 and EGFR, respectively, was shown. hTM and CM IgA2 resulted in reduced proliferation of 59% and 56%, respectively. Irrelevant hOM IgA2 matched isotype negative control antibody did not affect the proliferation of SK-BR-3 or A-431 target cells. The antimitotic agent aphidicolin served as the positive control; in the presence of 10 μM aphidicolin, less than 15% target cell proliferation was observed (Figure 6). 

#### 3.3.2. IgA Antibodies Mediate Antibody-Dependent Cellular Cytotoxicity 

While proliferation inhibition is mediated by the interaction of the Fab domain of an antibody with its target on the target cell surface, antibody-dependent cellular cytotoxicity (ADCC) is mediated by the Fc part of an antibody when the antibody is bound to a target cell. ADCC is an important effector function of therapeutic antibodies for cancer therapy [58]. Immune effector cells are recruited by antibodies to elicit a cytotoxic effect on cancer cells. The antibody Fab domain specifically binds to its target on a cancer cell and thereby marks it for immune effector cells. Immune effector cells bind the Fc part of the antibody with Fc receptors. The presence of multiple targets and antibodies on the surface of a target cell results in crosslinking of Fc receptors, which in turn initiates intracellular activation of immune effector cells. 

Usually, NK cells, which are present in isolated peripheral blood mononuclear cells (PBMCs), serve as effector cells for in vitro evaluation of IgG antibodies [58]. In contrast to granulocytes, NK cells do not express FcαRI, which is required for IgA-dependent cellular cytotoxicity. Importantly, granulocytes constitute the most numerous leucocyte population in human blood [3,4,13]. Therefore, recruitment of granulocytes against cancer cells represents a promising strategy for cancer immunotherapy. The potential of IgA antibodies to activate granulocytes was shown (Supplementary A, Figure S7), and others used granulocytes as the effector cell to evaluate IgA antibodies’ biofunctionality [8,9,59]. Hence, granulocytes were used as effector cells to evaluate IgA-mediated cellular cytotoxicity against cancer cell lines. 

Granulocyte purity was assessed by flow cytometry and exceeded 95%. As determined by flow cytometry, FcαRI was abundantly expressed on purified granulocytes; 98% of granulocytes were positive. 

**Figure 7:**
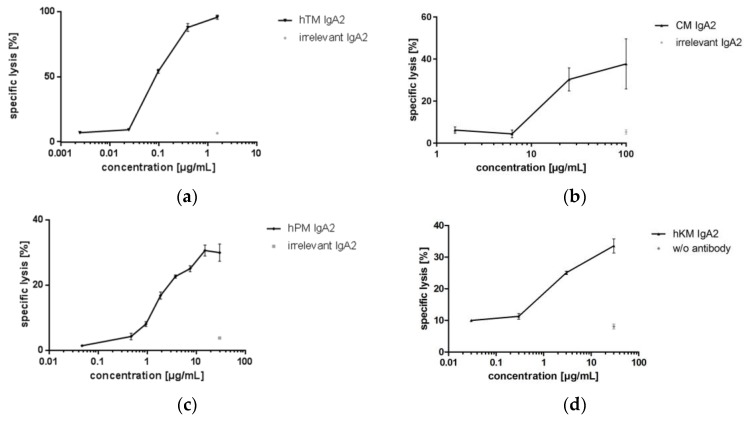
Antibody-dependent cellular cytotoxicity mediated by hTM, CM, hPM and hKM IgA2 antibodies against SK-BR-3, ZR-75-1 or Panc-1 target cells. Granulocytes isolated from peripheral blood were used as effector cells. Mean values (±SEM) of triplicates are shown. hTM (**a**) and CM IgA2 (**b**) mediated specific lysis of SK-BR-3 (Her2 +++, EGFR +) target cells. The irrelevant matched isotype control did not mediate lysis of SK-BR-3 target cells. hPM (**c**), and hKM IgA2 (**d**) mediated specific lysis of ZR-75-1 (TA-MUC1 +++) and Panc-1 (TF +) target cells, respectively. Antigen binding sites were determined using IgG antibodies and are indicated in brackets; antigen binding sites: +, 1 × 10^4^ to 1 × 10^5^; ++, 1 × 10^5^ to 5 × 10^5^; +++, >5 × 10^5^. One representative out of at least two independent experiments is shown.

Four IgA antibodies directed against different tumor-associated targets for solid cancer indications (Her2, EGFR, TA-MUC1, TF) were biofunctional in ADCC assays using granulocytes as effector cells and corresponding target cell lines. hTM and CM IgA2 antibodies mediated lysis of SK-BR-3 target cells. When comparing the two IgA antibodies directed against EGFR or Her2, CM IgA2 resulted in lower maximal lysis of SK-BR-3 target cells as compared to hTM IgA2, 31% and 89%, respectively (Figure 7a,b). In addition, higher antibody concentrations were required for mediating cellular cytotoxicity by CM IgA2 compared to hTM IgA2. This might be attributed to the higher Her2 antigen density as compared to EGFR antigen density on the surface of SK-BR-3 target cells. hPM IgA2 antibody concentration-dependent specific lysis of ZR-75-1 target cells was shown (Figure 7c). hKM IgA2 effectively mediated lysis of Panc-1 target cells at antibody concentrations exceeding 1 μg/mL (Figure 7d). Irrelevant IgA antibodies or medium without antibody served as negative controls, which did not mediate the lysis of target cells. 

#### 3.3.3. IgA Antibodies Mediate Antibody-Dependent Cellular Phagocytosis 

**Figure 8:**
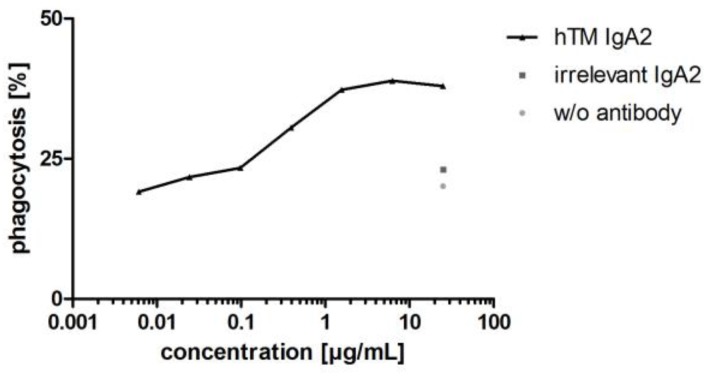
Antibody-dependent cellular phagocytosis (ADCP) mediated by hTM IgA2 antibodies against BT-474 target cells. Monocyte-derived macrophages (MDM) were used as effector cells for ADCP assays. Mean values of duplicates are shown. hTM IgA2 mediated phagocytosis of the BT-474 (Her2 +++) target cell line. hOM IgA2 irrelevant matched isotype or medium without antibody negative controls did not result in phagocytosis of target cells.

Antibodies targeting tumor antigens mediate phagocytosis of cancer cell lines by different effector cells including granulocytes, dendritic cells and macrophages [60,61,62]. In addition to granulocytes, macrophages represent an interesting effector cell population, which is found in large numbers within the tumor microenvironment [63]. Therefore, as a second Fc-mediated effector function for IgA antibodies, antibody-dependent cellular phagocytosis (ADCP) of cancer cell lines was investigated using MDM as effector cells. ADCP was shown for hTM IgA2 using BT-474 target cells and MDM as effector cells. hTM IgA2 mediated phagocytosis of BT-474 target cells. Maximal phagocytosis for hTM IgA2 was 39%. Unspecific phagocytosis for controls with irrelevant matched isotype control antibody or without antibody in the range of 5–20% was observed in ADCP assays (Figure 8).

As shown for hTM IgA2, IgA antibodies mediated phagocytosis of corresponding target cells. Apart from proliferation inhibition and ADCC, phagocytosis was shown as an additional mechanism of action of IgA antibodies directed against targets for solid cancer indications. 

#### 3.3.4. Biofunctionality of Anti-CD20 IgA2 Antibody 

**Figure 9:**
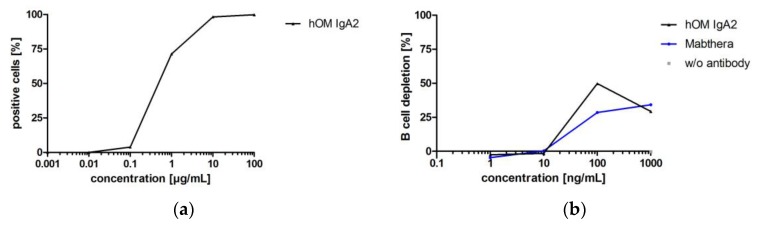
hOM anti-CD20 IgA2 antibody binds Raji target cells and mediates B cell depletion. (**a**) Concentration-dependent binding of hOM IgA2 to Raji target cells by flow cytometry. (**b**) Whole blood was used as a source for target and effector cells. Mean values of duplicates and one out of two independent experiments are shown. hOM IgA2 showed at least comparable maximal B cell depletion like commercially available Mabthera anti-CD20 IgG control.

While the antibodies investigated before for biofunctionality were directed against targets of solid cancer indications, hOM binds CD20, which is a target for hematological cancer indications. B cell depletion in whole blood was used to confirm the biofunctionality of hOM IgA2. Whether depletion of B cells in whole blood was triggered by Fab- or Fc-mediated effector functions could not be distinguished in this assay. However, this in vitro setup resembles the human situation because it includes all immunological components present in circulation [64]. Biofunctionality of hOM IgA2 was shown by flow cytometry and B cell depletion assays (Figure 9). hOM IgA2 showed maximal B cell depletion at about 100 ng/mL, while less B cell depletion was observed at 1000 ng/mL. Maximal B cell depletion for hOM IgA2 was at least comparable to commercially available Mabthera (Figure 9). 

## 4. Discussion

While monoclonal antibodies are established pharmaceuticals for cancer therapy, not all patients benefit from treatments [1,58]. In order to improve cancer therapy by increasing the functional diversity of antibodies, the aim of this study was to elucidate the potential to generate biofunctional IgA isotype antibodies as potential cancer therapeutics. For example, the interaction with other Fc receptors, which are expressed differently on the surface of immune effector cells, may improve efficacy. Several studies investigated anti-EGFR IgA antibodies [9,10,43,59,65]; yet, studies of IgA antibodies with other tumor-targeting specificities remain scarce, and no evaluation of a panel of IgA antibodies with multiple targets using a human expression system is available. IgA antibodies targeting Ep-CAM, human leukocyte antigen class II and CD20 were generated and functional against their target cells [4,5,66]. Bispecific formats against a tumor antigen and FcαRI were used to simulate IgA-mediated Fc-dependent effector functions against target cells [3,67,68,69]. Possibly due to the more difficult production and purification compared to IgG antibodies, until now, no IgA antibody has been clinically developed for cancer therapy. Here, a panel of monoclonal IgA antibodies targeting five heterogeneous antigens was successfully generated. Biochemical integrity was confirmed, and biofunctionalities against cancer cell lines were shown. 

### 4.1. Novel Tumor-Specific Monoclonal IgA Antibodies with Human Glycosylation 

Several expression systems have been used for the production of recombinant IgA antibodies: myeloma cells [70,71], monkey-derived COS cells [72], insect cells [73], baby hamster kidney cells [5] and plants [74,75]. However, most of the recombinantly-produced IgA antibodies were expressed in Chinese hamster ovary (CHO) cells [8,9,24,45,46,47,59,65,66,69,71,72,76,77]. The glycosylation of biotherapeutic biologicals is important for function, bioavailability and tolerance in human circulation. Non-human glycan structures attached to a therapeutic molecule can result in immunological reactions [26]. The functional role of IgA antibody *N*-glycans for Fc-mediated effector functions is controversial. However, the lack of carbohydrate moieties with adversely immunogenic potential in humans, such as α(1-3)-linked galactose and *N*-glycolylneuraminic acid, is desired, and the use of a human expression system is especially desired for molecules, which are highly glycosylated. Within their constant region of the heavy chain, IgA antibodies possess up to five *N*-glycosylation sites and in the case of IgA1 multiple *O*-glycosylation sites (Figure 1). In contrast, IgG antibodies currently approved for cancer immunotherapy, as well as other indications possess one *N*-glycosylation and no *O*-glycosylation sites in the constant region of the heavy chain. Moreover, glycan structures present in the Fc part of IgG antibodies are orientated towards the inter-HC space within the molecule, while it has been shown that IgA antibody glycans are orientated away from the molecule and surface accessible [24] (Figure 1). Immunological reactions towards non-human structures on therapeutics could result in neutralization, increased clearance or severe reactions upon exposure. Potential immunogenic carbohydrate structures on molecules produced in non-human expression systems include α(1-3)-linked galactose and *N*-glycolylneuraminic acid. In humans, glycans do not contain these structures or linkage-structure combinations [29]. In contrast, these carbohydrate moieties can be found on proteins from various rodent expression systems. In the case of mouse myeloma-derived monoclonal antibody Erbitux (cetuximab, Merck), in certain populations, the preexistence of IgE antibodies against the galactose-α(1-3)-galactose epitope present on the carbohydrate of Erbitux caused severe hypersensitivity reactions and anaphylactic shocks in patients [78]. Hence, using a human expression system like the mAbExpress GEX cell line for the production of IgA antibodies is even more important than for IgG antibodies because the number of glycosylation sites is not only much higher for IgA antibodies, but they are also orientated towards the outside of the molecule. Therefore, more potential immunogenic sites could be present, as well as accessible on therapeutic IgA antibodies depending on the expression system in which they are produced. Here, fully-human glycosylated recombinant monoclonal IgA antibodies directed against different cancer targets were successfully generated. Non-human glycan structures that could potentially be immunogenic were shown not to be present on recombinant IgA antibodies produced for this study in a GEX expression system. Moreover, sialylation is important to prolong IgA antibody serum half-life [20,43,65]. As compared to other reported *N*-glycosylation profiles, the generated IgA antibodies possessed much higher sialylation degrees. Boross et al. reported maximal sialylation degrees of 20% for the IgA antibodies expressed in CHO cells [43]. Brunke et al. reported 23% and no sialylated glycans for CHO-derived wild-type and stability-promoting mutated IgA2 antibodies, respectively [65]. *N*-glycosylation profiles of the IgA antibodies generated here revealed higher total sialylation degrees. Among the five analyzed IgA antibodies, more than 60% of complex type *N*-glycans were sialylated. Increased sialylation results in decreased clearance by the asialoglycoprotein receptor (ASGP-R) in the liver [43]. For human serum IgA1, 98% of *N*-glycans were reported to be sialylated [24]. However, due to clearance of less sialylated endogenous IgA antibodies, highly sialylated molecules might be enriched within circulation. The *N*-acetylneuraminic acid of sialylated IgA antibody *N*-glycans is mainly in α(2-6) configuration [24]. While CHO expression systems do not have the glycosyltransferase responsible for α(2-6)-linked *N*-acetylneuraminic acid [29], both α(2-3)- and α(2-6)-linked sialylated glycans can be found in GEX-derived products. 

Both, IgA1 and IgA2 with or without the J chain were successfully produced using the human good manufacturing practice (GMP)-validated expression system in serum-free conditions. One drawback of therapeutic IgA antibodies was the lack of established production systems, scale-up capabilities and purification strategies. Product heterogeneity might also arise due to the high number of glycosylation sites [8]. IgA productivity by a GEX cell line was high and allowed for fast generation of high yield monoclonal IgA antibody producing clones. In the past, specific production rates of up to 2.1 pg/cell/day [65], 0.8–5 pg/cell/day [8,46] or 0.17–16 pg/cell/day [47] were reported. Serum-free production of recombinant IgA antibodies has been described before [9]; maximal specific production rates were 2.2 pg/cell/day. Here, much higher production rates were achieved using serum-free conditions, up to 53 pg/cell/day for hTM IgA2 (Supplementary A, Table S1). Productivity differed between antibodies; CM IgA2 clones showed maximal specific production rates of 3 pg/cell/day, which was the lowest among the IgA antibodies generated. Similarly, IgG antibody productivities can differ, as well, which indicates that variable domains or transfection events affect productivity. Chromosomal localization of the target genes affects expression levels and could be responsible for varying production levels, as well [79,80]. In standard 1 L spinner cultures, high amounts of IgA antibody were produced (up to 56 mg). From our experiences with the GEX system, productivities could probably be further increased by extended clone development with additional amplifications and cloning rounds as usually done for therapeutic biologicals intended for GMP manufacture. 

As shown for hTM IgA2, productivity could be remarkably increased by cultivation in a 2 L bioreactor. Due to the controlled environment (pH, oxygen saturation, temperature, metabolite analysis) and constant fresh media supply within the perfusion process, much higher cell concentrations were reached as compared to spinner cultures. The bioreactor perfusion process with 1 L working volume greatly exceeded prior reported maximal yields of IgA production. On average over 35 days, 2200 mg were produced per week as compared to reported productivities of 2.5 mg per roller bottle per week or 5.9 mg per CL1000 production flasks per week [9,46,59]. The IgA production yield was at least comparable to IgG antibody GMP production processes using the GEX expression system. For the production of recombinant proteins, perfusion processes are advantageous compared to commonly-used fed-batch processes due to better quality of the produced molecules with respect to more completely processed *N*-glycans [81], as well as higher reproducibility from batch to batch and full scalability from 10 mL–1000 L bioreactors, which is our experience with the GEX system. In perfusion processes, product is constantly harvested from the reactor and does not accumulate during the process within the culture, which contains metabolites and cell debris as in the case of the fed-batch processes [82]. Cultivation of IgA2 producing cells using a continuous bioreactor process was achieved and confirms the feasibility of high yield production in a GMP environment as required for therapeutic antibodies intended for human use. 

### 4.2. Biochemical Integrity and Antigen Binding Characteristics 

Subsequent to production in serum-free media, single-step affinity chromatography yielded clean IgA preparations as shown by SDS-PAGE, Western blots and SEC. Biochemical analysis revealed high reproducibility for IgA productions and purifications, as well as preparations of IgA monomers in high purity by SEC which is necessary for the generation of homogenous biotherapeutics intended for human use. Monomer contents of affinity purified IgA antibodies lacking J chain co-expression and purity were comparable to the literature [9,10]. As shown here and by others [9,66,83] using SEC, IgA antibodies in the absence of the J chain form mainly monomers, while dimers and higher order multimers are present in lower abundance. By co-expression of the J chain, more dimers and multimers are formed. This was shown for all five IgA2 antibodies lacking J chain co-expression and two IgA2J antibodies co-expressed with the J chain. At mucosal sites, dimeric IgA is transported through epithelial cells into the luminal site by pIgR. While the role of the J chain for pIgR binding is not clear [84], it promotes the formation of dimers in IgA-producing cells and could thereby contribute to the transport of dimeric IgA to generate secretory IgA upon luminal release [14]. 

Comparable antigen binding for IgG and IgA antibody monomers directed against EGFR was shown. This is consistent with data found in the literature comparing anti-EGFR IgG and IgA antibodies [8]. Here, surface plasmon resonance analysis revealed similar antigen binding kinetics for monomeric IgA and IgG antibodies. This was shown for non-tumor-targeting IgA antibodies before [44,76,85]. Moreover, monomeric and dimeric IgA2 antibodies were compared in antigen binding kinetic analysis. As shown for CM, IgA2J dimers showed increased avidity compared to IgA2 monomers. Most likely, this can be attributed to increased valence with two and four antigen binding sites for monomeric and dimeric IgA2, respectively. In sensorgrams, the main difference was the decreased dissociation rate for IgA2 dimers compared with IgA2 monomers. Together, antigen binding experiments showed that binding characteristics and avidity are retained when switching form IgG to IgA isotype antibodies. However, compared to IgA monomers, IgA dimers with higher valence showed increased avidity. 

### 4.3. IgA Antibodies as a Novel Class for Cancer Immunotherapy: Modes of Action 

In order to evaluate IgA isotype antibodies as a potential novel class of tumor-specific immunotherapeutics, biofunctionalities against cancer cell lines were investigated. IgA antibodies inhibited the proliferation of cancer cells, mediated ADCC and ADCP. 

As shown for anti-EGFR IgA2 antibodies before [9,59], the generated IgA2 antibodies directed against EGFR or Her2 inhibit the proliferation of cancer cell lines. 

Cytotoxicity assays revealed potent lysis of target cells mediated by IgA antibodies when using granulocytes as a source for effector cells. Granulocytes were chosen as effector cells because they are the most numerous leucocyte population in human blood; they express FcαRI; and multiple studies have shown that IgA antibodies or FcαRI-engaging bispecific formats are more potent than IgG antibodies or FcγR-engaging bispecific formats [3,5,6,8,9,59,67,68]. IgA2 antibodies were described to be more effective than IgA1 antibodies as shown for anti-human leukocyte antigen and anti-EGFR IgA antibodies [5,8]. Binding experiments with IgA1 and IgA2 to FcαRI-transfected cells or primary granulocytes revealed increased binding for IgA2, which might be an explanation for the more potent ADCC of this subclass [8]. Moreover, studies with differently sialylated IgA1 and IgA2 revealed differences in FcαRI binding depending on subclass, as well as glycosylation [86]. However, the role of glycosylation for FcαRI binding remains controversial, while Basset et al. [86] found that glycosylation affects FcαRI binding; Mattu et al. [24] and Gomes et al. [87] showed that IgA glycosylation is not important for binding this receptor. Considering studies that showed no difference between IgA antibodies of various sources or PNGase F-treated IgA antibodies in FcαRI binding [87]; comparable ADCC potency might be expected for IgA antibodies produced in expression systems resulting in varying glycosylation patterns. Similarly, investigations using different recombinant IgA1 antibodies showed no dependency on glycosylation for FcαRI binding [24]. This is in contrast to IgG-IgG receptor interactions, where reduced core-fucosylated IgG Fc glycans and glycans with higher galactosylation (G2 structures) result in increased affinity towards Fcγ receptors and Fc-mediated functionality [31,88]. For defucosylated IgG, carbohydrate-carbohydrate interactions between receptor and antibody glycans are responsible for increased affinity and result in more potent FcγRIIIa-mediated ADCC [31]. As discussed before, IgA glycans may influence stability, as well as be involved in the interaction with microbes at mucosal sites [26]. Due to their surface-exposed orientation, glycosylation effects on solubility and pharmacodynamics through interaction with glycan-specific receptors or lectins relevant for serum half-life and distribution are expected to be more pronounced for IgA than for IgG antibodies. This highlights the importance of IgA glycosylation, even though it does not directly affect FcαRI-mediated effector functions. In conclusion, ADCC was successfully shown as the mode of action for all four IgA antibodies with targets of solid cancer indications. The more potent ADCC activity of hTM compered to CM IgA2 antibody was most likely attributed to the higher Her2 antigen density as compared to EGRF, because one factor influencing ADCC activity is antigen density [89]. 

As an additional Fc-mediated mode of action, the potential of IgA antibodies to mediate phagocytosis of cancer cell lines was shown using monocyte-derived macrophages as effector cells. Before, FcαRI-mediated phagocytosis has been shown using a bispecific antibody directed against FcαRI and Her2 [90]. Moreover, recombinant IgA2 directed against EGFR was investigated in macrophage-mediated ADCC assays [59]. Herein, phagocytosis was shown for a recombinant anti-Her2 IgA2 antibody. hTM IgA2 concentration-dependent phagocytosis was shown using a cancer cell line and monocyte-derived macrophages as effector cells. Hence, macrophages might represent another effector cell population for therapeutic IgA antibodies against cancer cells. As shown for different cancers, tumor-associated macrophages are involved in tumor vascularization [91] and constitute up to 50% of the cell mass of a tumor [63]. Certain macrophages in the tumor environment can act as negative suppressor cells for T cell-mediated anti-tumor activity, as well as the promotor of tumor progression and invasion [92]. Using therapeutic antibodies to mediate the phagocytic activity of this lymphocyte population within the tumor microenvironment and redirect macrophages against tumor cells could represent a promising effector functionality for cancer therapy. 

The CD20-specific hOM IgA2 was functional in target cell binding, as well as B cell depletion at rather low concentrations exceeding 10 ng/mL. Interestingly, at the highest hOM IgA2 concentration tested, a decrease of B cell depletion was observed. In molar excess of IgA over targets, this could indicate anti-inflammatory signaling during monovalent binding of free IgA antibodies to the FcαRI [17,36,37]. However, similar effects at high antibody concentrations were observed for ofatumumab, an anti-CD20 IgG antibody [64]. Saturation of both targets and Fc receptors might result in decreased functionality at high antibody concentrations; hence, dose finding tests of the functionality would be important for in vivo application. While B cell depletion using whole blood resembles the human situation for therapeutics very well, other functionalities like lysosomal membrane permeabilization or homotypic adhesion of target cells could be investigated for further characterization [93]. Biofunctionality of the generated anti-CD20 IgA2 antibody indicates that IgA isotype antibodies could also be applicable for hematological cancer indications [66]. 

Evaluation of therapeutic IgA antibodies in vivo is difficult because mice do not express FcαRI, which is responsible for cellular cytotoxicity mediated by IgA antibodies [94]. In xenograft models, efficacy would be related to direct Fab-mediated effector functions or transgenic animals with IgA-specific Fc receptors on the surface of immune effector cells would be required. Nevertheless, the difference between mice and humans in various Fc receptors complicates these matters and the conclusions to be deduced. Compared to IgG, an anti-EGFR IgA2 antibody showed comparable and better anti-tumor efficacy in transgenic mouse xenograft models [43]. Recently, protein and cell line engineering was used to decrease the number of glycosylation sites on IgA antibodies and increase the sialylation degree of glycans, respectively, and thereby improve pharmacokinetic properties and in vivo efficacy due to reduced terminal galactose [95,96,97]. Still, due to faster clearance, IgA2 antibodies were administered five to eleven times as compared to a single administration of IgG antibodies at equal doses. With more frequent administration and protein or cell line engineering, IgA2 antibodies reached comparable tumor growth inhibition like IgG antibodies [96,97]. By the generation of non-engineered IgA antibodies with completely sialylated *N*-glycans, in vivo efficacy could potentially be further increased because of better pharmacokinetic behavior. This could be established with new higher and maximal sialylation cell lines related to mAbExpress, which recently became available in the GEX system. In addition, while CHO-derived *N*-glycans exclusively possess α2,3-linked sialic acid, the majority of human serum IgA is linked in α2,6 configuration [24,29]. Whether the human glycosylation pattern of the generated IgA antibodies translates into better in vivo pharmacokinetic behavior and/or efficacy remains to be elucidated. 

The potential of IgA antibodies for cancer therapy is also supported by data showing that, apart from other isotypes, production of IgA antibodies was induced after vaccination with recombinant carcinoembryonic antigen protein [98]. Moreover, immunohistochemically staining of primary human breast cancer samples showed high abundance IgA1 antibodies on the membrane and in the cytosol [99]. Taken together, there are still challenges remaining to elucidate the potential of IgA antibodies in vivo, but involvement of IgA isotype antibodies in human cancer has been shown. 

## 5. Conclusions

IgA antibodies against five targets were successfully generated and biofunctional against cancer cell lines. Large quantities and high quality monoclonal IgA antibodies were expressed. Hence, therapeutic IgA antibodies could be produced in sufficient amounts for potential application to treat cancer. We described how challenges associated with the production of these heavily glycosylated antibodies could be overcome. The IgA antibodies possessed human glycosylation patterns, which is especially desired for IgA *N*-glycans, which are more numerus and surface exposed compared to IgG *N*-glycans. To generate more affine dimeric IgA antibodies, J chain co-expression was successful to accomplish increased dimer association. Both, potent Fab- and Fc-mediated functionalities against cancer cell lines were shown. However, whether in vitro activity translates into tumor growth inhibition or cytotoxicity against cancer in patients remains to be elucidated. In this study, the in vitro activity of IgA antibodies varied depending on the target and tested cancer cell line. In line with attempts to personalize medicinal treatments [100], it might be feasible to search for and define biomarkers that could indicate beneficial therapeutic potential of one or another target for a patient. Moreover, increasing the functional diversity of cancer therapeutics with isotypes other than IgG could increase therapeutic options [101,102]. Patients might benefit from IgA antibody therapy where IgG antibodies lack efficacy and thereby represent a promising strategy for cancer therapy. 

## Figures and Tables

**Table 1:** Overview of the targets, isotypes and production levels of the generated IgA antibodies.

Name (Abbreviation)	Target	Variants Generated	Titer (mg/L)
PankoMab (hPM)	Tumor-associated mucin 1 (TA-MUC1)	IgA1	41.8
		IgA2	4.0 *
TrasGEX (hTM)	Epidermal growth factor receptor 2 (Her2)	IgA2	55.8
		IgA2J	41.5
CetuGEX (CM)	Epidermal growth factor receptor 1 (EGFR)	IgA2	6.5
		IgA2J	9.8
KaroMab (hKM)	Thomsen-Friedenreich (TF) antigen, core 1	IgA2	24.7
ObiGEX (hOM)	B-lymphocyte antigen CD20	IgA2	15.3

Names were introduced for IgG antibodies; mean titer of 2–5 clones in supernatants of spinner cultures upon harvest; * one selected clone.

**Table 2:** Relative abundance of glycan parameters for complex-type *N*-glycans.

IgA2	Relative Molar Amount of Glycan Parameters (%)							
Antibody	F	B	S0	S > 0	G0	G > 0	αGal	NeuGc
hTM	64	15	32	62	3	91	0	0
hOM	79	9	29	64	11	81	0	0
hPM	82	12	29	68	4	92	0	0
CM	70	12	27	63	10	81	0	0
hKM	84	7	34	62	12	83	0	0
IgG Erbitux	85	0	81	16	30	66	28	15

F: glycans with fucose; B: glycans with bisecting *N*-acetylglucosamine; S: glycans with *N*-acetyl neuraminic acid; G: glycans with galactose, αGal: glycans with galactose α(1-3)-galactose (Galili epitope); NeuGc: glycans with *N*-glycolylneuraminic acid; numbers indicate monomer units per *N*-glycan.

## Supplementary Materials

The following are available online at http://www.mdpi.com/2306-5354/4/2/42/s1: Supplementary A: Results; Supplementary B: Material and Methods.
