# Supplementary material for: Human Cell Line-Derived Monoclonal IgA Antibodies for Cancer Immunotherapy
Source: Bioengineering (Basel). 2017 May 8;4(2):42. doi: 10.3390/bioengineering4020042 (PMC5590476; doi:10.3390/bioengineering4020042)
Supplement: Supplementary file 1 [file bioengineering-04-00042-s001.pdf]

Supplementary Materials:

Section A – Results

Specific production rates

Table A1. IgA antibody production in selected mAbExpress clones.

Antibody	q(mAb) [pg/cell/day]
hTM IgA2	53.3 ±12.4
hTM IgA2J	19.5 ±5.4
hPM IgA1	10.9 ±1.3
hKM IgA2	11.9 ±4.5
hOM IgA2	n.d.
CM IgA2J	3.6 ±0.6
CM IgA2	2.4 ±0.5
hPM IgA2	4.6 ±2.3

q(mAb): specific production rate for 4 to 5 selected clones; mean ± standard deviation; n.d.: not determined

Analytical size exclusion chromatography

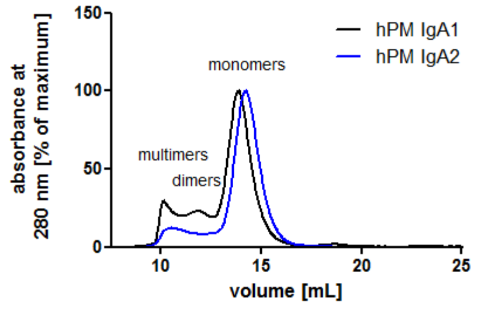

(a)

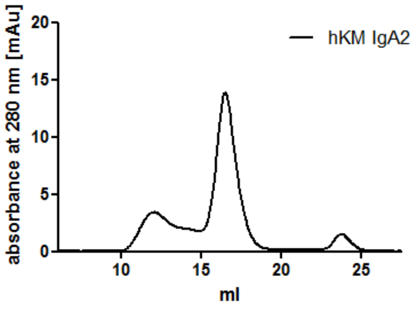

(b)

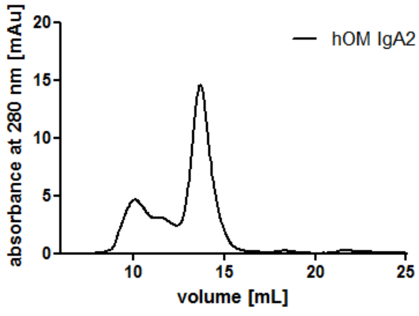

(c)

Figure A1. Analysis of hPM IgA1 and IgA2 **(a)** , hKM IgA2 **(b)** and hOM IgA2 **(c)** by size exclusion chromatography. Affinity purified IgA antibodies showed comparable profiles in SEC. The presence of fragments is restricted to hKM IgA2 and hTM IgA2 (main text, Figure 5a).

CM IgA2J dimer preparation by size exclusion chromatography

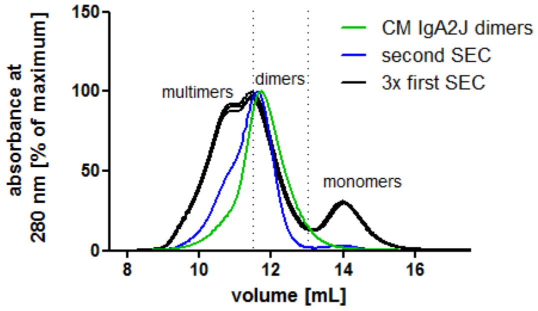

Figure A2. Preparation of CM IgA2J dimers by size exclusion chromatography. CM IgA2J dimers were generated in two subsequent preparative SECs by fractionating the right side of the dimer peak (vertical dotted lines). The first SEC was conducted three times to obtain sufficient material for the second SEC. Clean CM IgA2J dimers were generated as analyzed by additional analytical SEC (green).

Antigen and target cell binding

(a)

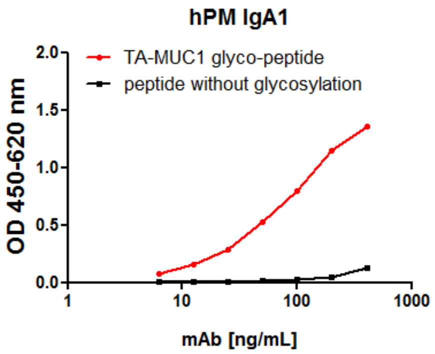

(b)

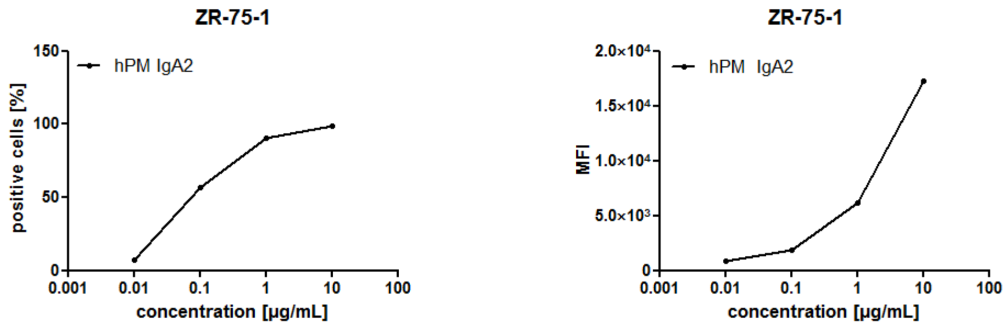

Figure A3. Specific antigen binding and ZR-75-1 target cell binding of hPM IgA antibodies were confirmed by enzyme-linked immunosorbent assay (ELISA) and flow cytometry. **(a)** Antigen ELISA using TA-MUC1 peptide (red) and non-glycosylated MUC1 peptide as negative control (black). hPM IgA1 specifically binds to the glycosylated TA-MUC1 peptide, less than 10% binding signal for the non-glycosylated peptide at the highest concentration tested. **(b)** Concentration-dependent binding of hPM IgA2 to ZR-75-1 target cells was shown as percent positive cells (left) and median fluorescence intensity (MFI, right). Mean values of duplicates are shown. One exemplary out of at least two independent experiments is shown.

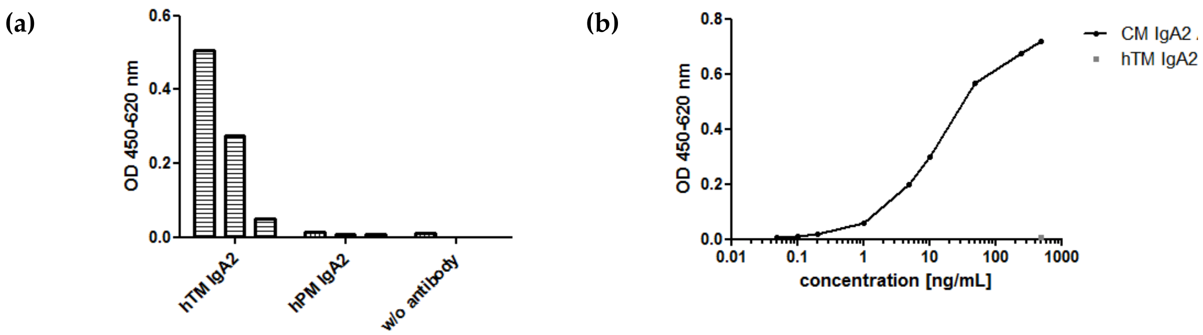

Figure A4. Antigen binding of hTM IgA2 and CM IgA2 was shown by enzyme-linked immunosorbent assay (ELISA). Her2 and EGFR were coated on ELISA plates to investigate hTM IgA2 and CM IgA2 binding, respectively. **(a)** Transiently expressed hTM IgA2 was tested for antigen binding to Her2. Three dilutions (undiluted, 1:10 and 1:100 dilution from left to right) of non-purified supernatant samples from cells expressing hTM IgA2 and hPM IgA2 were used. Concentration-dependent binding of hTM IgA2 to Her2 was confirmed. Irrelevant matched isotype hPM IgA2 and undiluted supernatant without antibody negative controls did not bind. **(b)** For CM IgA2, antigen binding was confirmed for purified antibody. Irrelevant matched isotype hTM IgA2 served as negative control. Mean values of duplicates are shown.

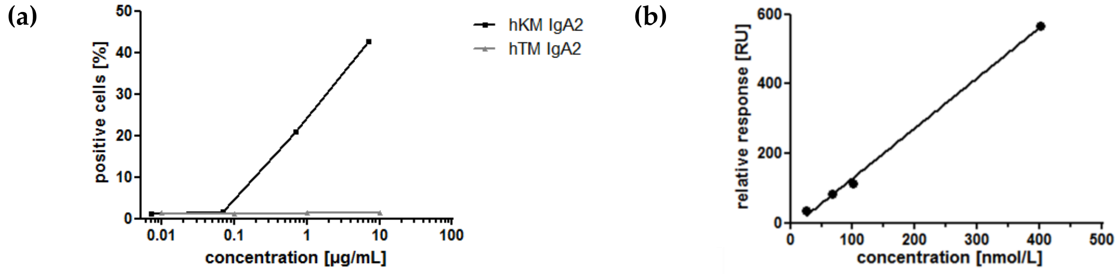

Figure A5. Binding of hKM IgA2 to Raji target cells and immobilized asialoglycophorin (AGP) by flow cytometry and surface plasmon resonance, respectively. **(a)** Unpurified supernatant of hKM IgA2 antibody producing cell culture was used in flow cytometry experiments. IgA antibody concentrations were determined by titer enzyme-linked immunosorbent assay and purified hTM IgA2 antibody served as negative control. Mean values of duplicates are shown. **(b)** AGP carrying the TF antigen was immobilized on one flow cell of a CM5 chip and a second flow cell served as reference (as described for EGFR). Varying concentrations of purified hKM IgA2 monomers were injected at a flow rate of 10 µL/min for 2 min association time and followed by 5 min dissociation time. Concentration-dependent biding was illustrated by relative maximal response units as a function of hKM IgA2 antibody concentration. One exemplary out of two independent SPR experiments.

FcαRI binding

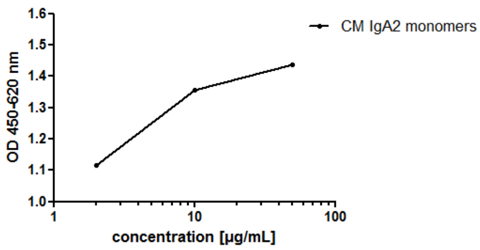

Figure A6. Specific binding of IgA antibody to FcαRI extracellular domain. Mean values of duplicates are shown. One exemplary out of at least two independent experiments is shown. CM IgA2 monomers binding to recombinant FcαRI was shown by enzyme-linked immunosorbent assay (ELISA).

IgA antibodies activate effector cells in the presence of target cells

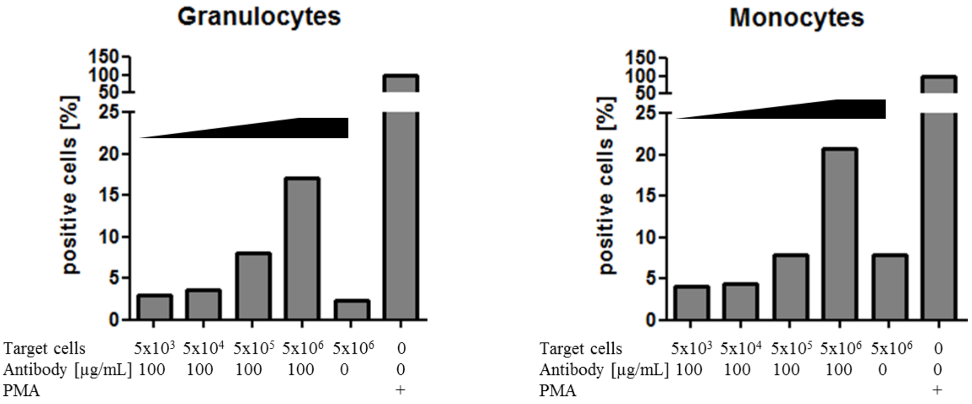

Figure A7. IgA antibody-mediated activation of granulocytes and monocytes in the presence of target cells. Mean values of duplicates are shown. One exemplary out of two independent experiments is shown. Antibodies and target cells were added to whole blood to investigate the potential of IgA antibodies to mediate effector cell activation. Activation was investigated by measuring the induction of reactive oxygen species (ROS) by flow cytometry. Addition of hPM IgA1 antibody and ZR-75-1 target cells in whole blood resulted in activation of granulocytes and monocytes. With increasing target cell concentrations (indicated by black bar) more granulocytes and monocytes were activated. In the absence of antibody, granulocyte and monocyte activation was low. Granulocytes and monocytes were distinguished by flow cytometry with corresponding gates in forward versus sideward scatter plots. Phorbol 12-myristate 13-acetate (PMA) served as positive control for granulocyte and monocyte activation (+).

Section B – Material and Methods

Enzyme-linked immunosorbent assays (ELISAs)

Antibodies specific for antibody isotypes, antigens or antibodies specific for a tagged receptor (see below) were coated overnight on Maxisorp 96-well plates (Nunc) at a concentration of 0.5 µg/mL to 1 µg/mL. Serial dilutions of antibody samples were prepared and isotype specific HRP-conjugated antibodies were used for detection. ELISAs were conducted according to standard protocols [S1].

IgA antibody concentrations in supernatants were determined using the human IgA ELISA Quantitation Set (Bethyl) according to the manufacturer's protocol.

In case of hPM, a synthetic TA-MUC1 glycosylated peptide was used as antigen. To confirm the mixed glycan-peptide epitope, a non-glycosylated peptide served as negative control. The assay was described previously by Danielczyk et al. [S2].

For FcαRI ELISA, after blocking the plate, each well was incubated with a constant concentration of His-tagged recombinant CD89 prior to sample incubation.

ELISAs were developed using TMB microwell substrate solution (Tebu-bio) and sulfuric acid was used to stop the reaction. For detection at 450 nm and 620 nm an Infinite F200 microplate reader (Tecan) was used.

Determination of the specific production rate

Clones were screened for antibody production per cell per day. Washed cells (4x10^4 cells/mL) were seeded in 0.5 mL medium without selection pressure in 24-well plates. After 3 or 4 days incubation, the volume of the cell suspension was measured, cells were counted and supernatant samples were taken for IgA quantification ELISA (Bethyl). The specific production rate was calculated under the assumption of exponential growth during the 3 or 4 day incubation time using the following formula:

specific production rate [pg/cell/day] = $titer_{antibody}$ [pg/mL]/integral cell area,

where

integral cell area [cell x d/mL] = (final cell count [cells/mL] – $4x10^4$ cells/mL)/log_e(final cell count [cells/mL]/$4x10^4$ cells/mL) x time of culture [d].

Equations for SPR models

Bivalent

A = Conc, B[0] = RMax

dB/dt = - (ka1*A*B - kd1*AB) - (ka2*AB*B - kd2*AB2)

AB[0] = 0

dAB/dt = (ka1*A*B - kd1*AB) - (ka2*AB*B - kd2*AB2)

AB2[0] = 0

dAB2/dt = (ka2*AB*B - kd2*AB2)

Total response: AB + AB2 + RI

Tetravalent

A = Conc; B[0] = RMax

dB/dt = - (ka1*A*B - kd1*AB) - (ka2*AB*B - kd2*AB2) - (ka3*AB2*B - kd3*AB3) - (ka4*AB3*B - kd4*AB4)

AB[0] = 0

dAB/dt = (ka1*A*B - kd1*AB) - (ka2*AB*B - kd2*AB2)

AB2[0] = 0

dAB2/dt = (ka2*AB*B - kd2*AB2) - (ka3*AB2*B - kd3*AB3)

AB3[0] = 0

dAB3/dt = (ka3*AB2*B - kd3*AB3) - (ka4*AB3*B - kd4*AB4)

AB4[0] = 0

dAB4/dt = (ka4*AB3*B - kd4*AB4)

Total response: AB + AB2 + AB3 + AB4 + RI

Respiratory burst assay

The Phagoburst kit (Glycotope Biotechnology, Heidelberg, Germany) was used for the respiratory bust assay. The manufacturer's protocol was adapted by the addition of target cells to investigate the dependency on target cells for activation of granulocytes and monocytes.

Antibodies, peptides and proteins

ELISA and Western blot antibodies (concentrations or dilutions are indicated)

Anti-alpha chain from IgA Quantitation Kit (1:100 for Western blots)	E80-102	Bethyl Laboratories (Montgomery, USA)
Anti-goat IgG (1:2,000)	705036	Jackson ImmunoResearch (West Grove, USA)

Anti-human IgA horseradish peroxidase (POD) (1:5,000)	309-035-011	Jackson ImmunoResearch
Anti-human IgG POD (1:10,000)	109-035-098	Jackson ImmunoResearch
Anti-J chain (5 µg/mL)	C58617	LifeSpan Biosciences (Seattle, USA)
Anti-kappa LC (1µg/mL)	ab1050	Abcam (Cambridge, UK)
Anti-mouse IgG POD (1:2,000)	P0447	Dako (Glostrup, Denmark)
Anti-Tetra His (1 µg/mL)	LS-C15474	LifeSpan Biosciences
Flow cytometry antibodies (dilutions are indicated or 5 µL per 100 µL cell suspension were used)		
Anti-CD3 fluorescein isothiocyanate (FITC)	555332	BD Biosciences (San Jose, USA)
Anti-CD19 allophycocyanin (APC)	555415	BD Biosciences
Anti-CD89 PE	555686	BD Biosciences
Anti-human IgA Cyanine (Cy) 3 (1:200)	109-166-011	Jackson ImmunoResearch
Anti-human IgG Cy3 (1:200)	109-165-098	Jackson ImmunoResearch
Mouse IgG1 AlexaFluor 647	557714	BD Biosciences
Mouse IgG1 FITC	555748	BD Biosciences
Mouse IgG1 PE	555749	BD Biosciences
Recombinant proteins, synthetic peptides and reference antibody		
EGFR (antigen ELISA)	E3641	Sigma-Aldrich (St. Louis, USA)
EGFR (SPR)	344-ER	R&D Systems (Minneapolis, USA)
Her2 extracellular domain GST	internal by Lead Discovery Department Glycotope	
Asialoglycophorin A	A9791	Sigma-Aldrich
Custom synthetic MUC1 peptides	as described before [S2]	
CD89	YSP1147	Speed Biosystems (Gaithersburg, USA)
MabThera, rituximab		Roche (Welwyn Garden City, UK)

Amino acid sequences of generated antibodies

	Uniprot ID/reference

Ig alpha-1 chain C region	P01876
Ig alpha-2 chain C region	P01877
Joining chain	P01591
Ig kappa chain C region	P01834
Ig gamma-1 chain C region	P01857
hPM variable domains	as described before [S2]
hTM variable domains	www.drugbank.ca (Acession Number DB00072)
CM variable domains	www.drugbank.ca (Acession Number DB00002)
hKM variable domains	humanized Nemod-TF2 [S3]
hOM variable domains	www.drugbank.ca (Acession Number DB08935)

Consumables

Cell culture dishes, centrifuge tubes, bottle-top filter (TPP), serological pipets (Greiner Bio One), pipette tips (Axygen Scientific and Gilson), white flat-bottom 96-well plates (Costar), electroporation cuvettes (Amaxa and Eppendorf), nitrocellulose membrane (GE Healthcare, Little Chalfont, UK), gels for SDS-PAGE (Bio-Rad), protein solution concentrators, sterile filter (Merck Millipore), syringes and needles (B. Braun, Melsungen, Germany).

Eukaryotic cell lines – media composition, sources and media supplements

Cell lines, media composition	Source
A-431, DMEM, 10% FBS, 4 mM L-glutamine	DSMZ no. ACC 91 (Brunswick, Germany)
BT-474, HybriCare, 10% FBS, 2 mM L-glutamine	DSMZ no. ACC 64
GlycoExpess mAbExpress, Glycotope medium	Glycotope, Berlin, Germany
Panc-1, DMEM, 10% FBS, 4 mM L-glutamine	DSMZ no. ACC 783
Raji, RPMI 1640, 10% FBS, 2 mM L-glutamine	DSMZ no. ACC 319
SK-BR-3, McCoy's 5A, 10% FBS	Cell Line Service No 300333
ZR-75-1, RPMI 1640, 10% FBS, 2 mM L-glutamine	ATCC No CRL 1500

Media, supplements and additives

Accutase	L11-007, GE Healthcare (Little Chalfont, UK)
Dulbecco's MEM (DMEM)	F0415, Biochrom (Berlin, Germany)
Fetal Bovine Serum (FBS)	S0115, Biochrom

G 418 Sulfate	345812, Merck Millipore (Billerica, USA)
Glycotope medium	custom formulation, Biochrom
HybriCare	50188277FP, Fisher Scientific (Pittsburgh, USA)
L-glutamine, 200 mM	K0283, Biochrom
McCoy's 5A (Modified) Medium	26600, Thermo Fisher Scientific (Waltham, USA)
Methotrexate hydrate	M8407, Sigma-Adrich
Puromycin	631306, Clontech (Mountain View, USA)
RPMI 1640	F1215, Biochrom
Trypsin-EDTA	25200, Thermo Fisher Scientific (Waltham, USA)

References

[S1] J. Sambrook and D. W. Russell, *Molecular Cloning: A Laboratory Manual, Volume 1*. CSHL Press, 2001.

[S2] A. Danielczyk, R. Stahn, D. Faulstich, A. Löffler, A. Märten, U. Karsten, and S. Goletz, “PankoMab: A potent new generation anti-tumour MUC1 antibody,” *Cancer Immunol. Immunother.*, vol. 55, no. 11, pp. 1337–1347, 2006.

[S3] S. Goletz, Y. Cao, A. Danielczyk, P. Ravn, U. Schoeber, and U. Karsten, “Thomsen-Friedenreich antigen: the ‘hidden’ tumor antigen.,” *Adv. Exp. Med. Biol.*, vol. 535, pp. 147–62, Jan. 2003.
